# Cell type mapping of inflammatory muscle diseases highlights selective myofiber vulnerability in inclusion body myositis

**DOI:** 10.1038/s43587-024-00645-9

**Published:** 2024-06-04

**Authors:** Sven Wischnewski, Thomas Thäwel, Chiseko Ikenaga, Anna Kocharyan, Celia Lerma-Martin, Amel Zulji, Hans-Werner Rausch, David Brenner, Leonie Thomas, Michael Kutza, Brittney Wick, Tim Trobisch, Corinna Preusse, Maximilian Haeussler, Jan Leipe, Albert Ludolph, Angela Rosenbohm, Ahmet Hoke, Michael Platten, Jochen H. Weishaupt, Clemens J. Sommer, Werner Stenzel, Thomas E. Lloyd, Lucas Schirmer

**Affiliations:** 1 Department of Neurology, Medical Faculty Mannheim, Heidelberg University, Mannheim, Germany; 2 Department of Neurology, Johns Hopkins University School of Medicine, Baltimore, MD USA; 3 Department of Neurology, University of Ulm, Ulm, Germany; 4 Genomics Institute, University of California, Santa Cruz, Santa Cruz, CA USA; 5 Department of Neuropathology, Charité-Universitätsmedizin Berlin, Corporate Member of Freie Universität Berlin, Humboldt-Universität zu Berlin and Berlin Institute of Health, Berlin, Germany; 6 Division of Rheumatology, Department of Medicine V, Medical Faculty Mannheim, Heidelberg University, Mannheim, Germany; 7 Deutsches Zentrum für Neurodegenerative Erkrankungen, Ulm, Germany; 8 Solomon H. Snyder Department of Neuroscience, Johns Hopkins University School of Medicine, Baltimore, MD USA; 9 DKTK Clinical Cooperation Unit Neuroimmunology and Brain Tumor Immunology, German Cancer Research Center, Heidelberg, Germany; 10 Mannheim Center for Translational Neuroscience, Medical Faculty Mannheim, Heidelberg University, Mannheim, Germany; 11 Mannheim Institute for Innate Immunoscience, Medical Faculty Mannheim, Heidelberg University, Mannheim, Germany; 12 Interdisciplinary Center for Neurosciences, Heidelberg University, Heidelberg, Germany; 13 Institute for Neuropathology, University Medical Center, Johannes Gutenberg-University Mainz, Mainz, Germany; 14 Department of Neurology, Baylor College of Medicine, Houston, TX USA

**Keywords:** Neuromuscular disease, Neuroimmunology, Ageing

## Abstract

Inclusion body myositis (IBM) is the most prevalent inflammatory muscle disease in older adults with no effective therapy available. In contrast to other inflammatory myopathies such as subacute, immune-mediated necrotizing myopathy (IMNM), IBM follows a chronic disease course with both inflammatory and degenerative features of pathology. Moreover, causal factors and molecular drivers of IBM progression are largely unknown. Therefore, we paired single-nucleus RNA sequencing with spatial transcriptomics from patient muscle biopsies to map cell-type-specific drivers underlying IBM pathogenesis compared with IMNM muscles and noninflammatory skeletal muscle samples. In IBM muscles, we observed a selective loss of type 2 myonuclei paralleled by increased levels of cytotoxic T and conventional type 1 dendritic cells. IBM myofibers were characterized by either upregulation of cell stress markers featuring *GADD45A* and *NORAD* or protein degradation markers including RNF7 associated with p62 aggregates. *GADD45A* upregulation was preferentially seen in type 2A myofibers associated with severe tissue inflammation. We also noted IBM-specific upregulation of *ACHE* encoding acetylcholinesterase, which can be regulated by *NORAD* activity and result in functional denervation of myofibers. Our results provide promising insights into possible mechanisms of myofiber degeneration in IBM and suggest a selective type 2 fiber vulnerability linked to genomic stress and denervation pathways.

## Abstract (web summary)

Inclusion body myositis (IBM) is a progressive inflammatory muscle disease of unknown cause, prevalent in older adults. Through spatial and single nuclear profiling, the authors identify a selective type 2 myofiber pathology in IBM, linked to genomic stress and denervation.

## Main

IBM is a chronic and slowly progressive idiopathic inflammatory myopathy (IIM) with additional degenerative features. Muscle pathology in IBM is characterized by endomysial, highly differentiated T cell infiltrates, accumulation of ubiquitinated protein aggregates, rimmed vacuoles and mitochondrial damage^1^. Conversely, IMNM is a subacute IIM characterized by diffusely distributed necrotic myofibers infiltrated primarily by macrophages^2^. As the pathogenesis of IBM remains enigmatic with no effective therapy available, there is a high need to understand the mechanisms underlying progressive tissue damage and identify therapeutic targets. IBM is the most common IIM in older adults^1^ and, probably, will be even more prevalent in the future with an aging population.

In the present study, we focused on quadriceps femoris muscles and conducted a paired single-nucleus RNA sequencing (snRNA-seq) and spatial transcriptomics (ST) study. Using a combination of unsupervised computational methods in combination with in situ marker validation, we mapped the cell-type-specific drivers underlying muscle inflammation and progressive pathology in IBM in contrast to IMNM and noninflammatory skeletal muscle.

We could identify all major cell types present in noninflammatory and inflamed human skeletal muscles and mapped their transcriptomic signatures to the local tissue environment. In IBM, the inflamed tissue environment was characterized by increased densities of cytotoxic T lymphocytes (CTLs) and conventional type 1 dendritic cells (cDC1 cells). Homeostatic tissue macrophages, conversely, were relatively reduced in IBM muscle. Furthermore, we observed a selective loss of type 2 myonuclei (MNs) and a reduction in the tissue compartment corresponding to type 2 myofibers in IBM. Specifically, we could demonstrate that a subset of damaged IBM myofibers converged on cell/genomic stress pathways featuring genes such as *GADD45A* and the long noncoding RNA (lncRNA) *NORAD*, with *GADD45A* regularly found in type 2A fibers and linked to T cell infiltrates. Other IBM myofibers were characterized by the presence of protein degradation/autophagy pathway markers, including RNF7 associated with p62 protein aggregates. Finally, we noted IBM-specific myofiber upregulation of *ACHE* encoding acetylcholinesterase, which has been shown to be regulated via *NORAD* function and, therefore, could result in a functional denervation of damaged myofibers. Hence, our results suggest a selective type 2 fiber vulnerability in IBM linked to genomic stress pathways and functional denervation.

## Results

### Selective loss of type 2 MNs in inflamed IBM muscles

**Fig. 1:**
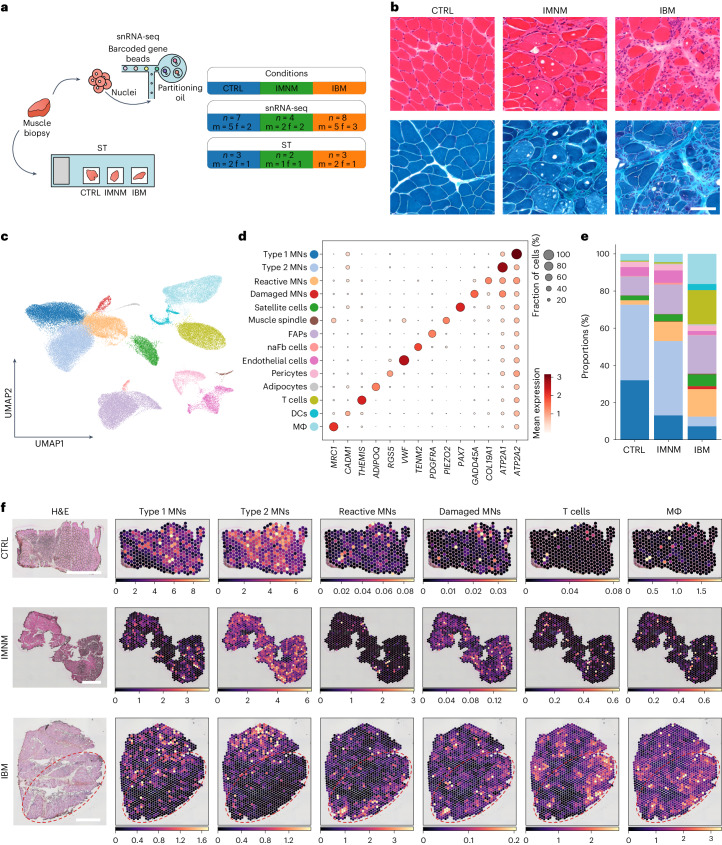
Overview of histology, snRNA-seq and ST. **a**, Sketch showing experimental design and information on the different groups of patients. **b**, Histology (H&E and Gömöri’s trichrome) showing representative images of CTRL, IMNM and IBM muscle. Scale bar, 100 µm. **c**, UMAP visualization of all cells/nuclei present in the snRNA-seq dataset from all conditions (CTRL, *n* = 7; IMNM, *n* = 4; IBM, *n* = 8; nuclei, *n* = 93,345). **d**, Dot plot showing marker genes of identified clusters. **e**, Compositional analysis of snRNA-seq data of CTRL, IMNM and IBM muscle (CTRL, *n* = 7; IMNM, *n* = 4; IBM, *n* = 8). Note, there are substantially increased immune cell populations and a decreased type 2 MN population in IBM. **f**, Representative images of deconvolution of ST data (CTRLs, *n* = 3; IMNM, *n* = 2; IBM, *n* = 3) showing locations of cell types identified in snRNA-seq of CTRL, IMNM and IBM muscle. Note, red contours highlight areas with low abundance of type II MNs and high abundance of immune cells based on gene expression in IBM. Scale bars, 1 mm. f, female; m, male.

**Extended Data Fig. 1:**
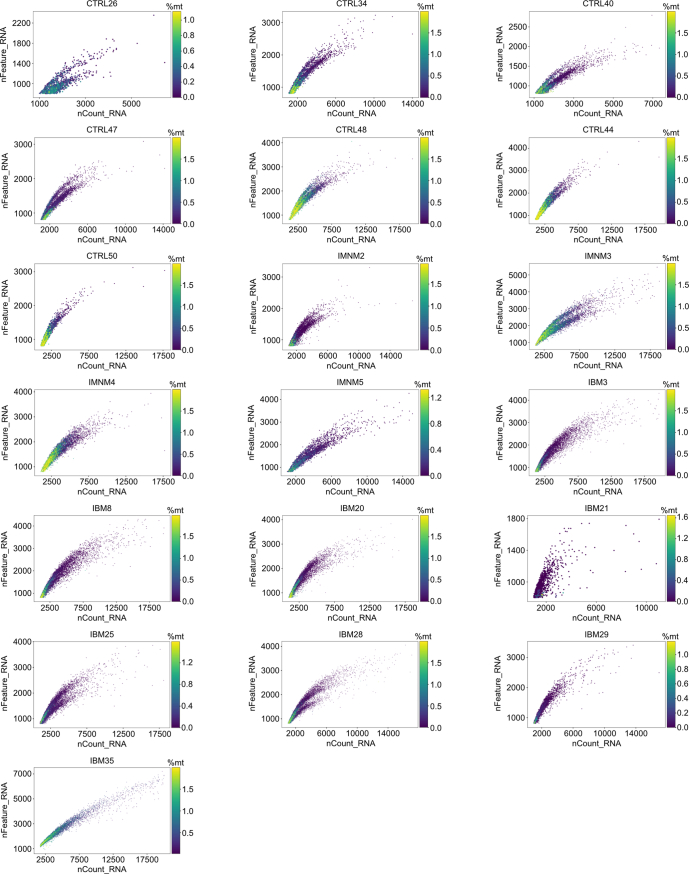
Quality control metrics in snRNA-seq analysis. Scatter plots showing quality control (QC) metrics nFeature_RNA, nCount_RNA, and percentages of mitochondrial encoded genes (%mt), after quality control of every sample included in our snRNA-seq analysis.

**Extended Data Fig. 2:**
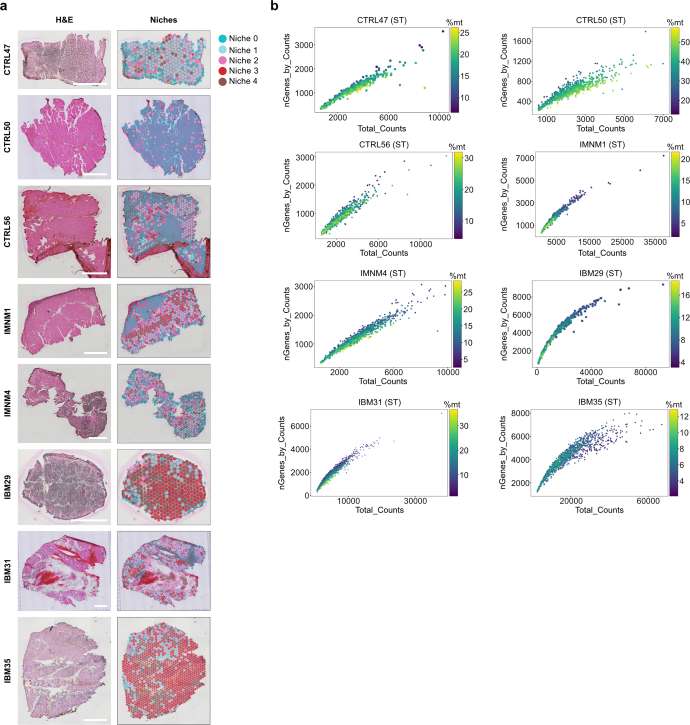
Quality control metrics in ST analysis. **a** Left column showing H&E histology of the tissue section used for ST. Right column showing the distribution of tissue niches on all sequenced sections. Scale bars = 1 mm. **b** Scatter plots showing QC metrics nGenes_by_Counts, Total_Counts, and percentages of mitochondrial encoded genes (%mt) for included spatial transcriptomic samples.

We performed snRNA-seq on seven noninflammatory controls (CTRLs), four IMNM and eight IBM quadriceps muscle biopsies, resulting in a total of 93,345 sequenced nuclei remaining after quality control (QC) (Fig. 1a,b and Extended Data Fig. 1). In addition, we performed ST from both paired and additional tissue samples (Fig. 1a and Supplementary Tables 1 and 2) comprising 3 CTRL, 2 IMNM and 3 IBM muscle biopsies, with a total of 7,462 sequenced spots remaining after QC (Extended Data Fig. 2a,b). When including all cell type profiles based on snRNA-seq and marker gene expression, we could identify MNs from type 1 fibers (*ATP2A2*, referred to here as type 1 MNs) and type 2 fibers (*ATP2A1*, type 2 MNs), reactive (*COL19A1*) and damaged MNs (*GADD45A*), and satellite cells (*PAX7*). Furthermore, we identified muscle spindle cells (*PIEZO2*)^3^, endothelial cells (*VWF*), pericytes (*RGS5*), adipocytes (*ADIPOQ*), one population of fibro-adipogenic progenitor (FAP) cells (*PDGFRA*) and one population probably representing nerve-associated fibroblasts (naFb cells) owing to the expression of genes related to synaptogenesis and nerve fiber development, for example, *NLGN1* and *TENM2* (refs. ^4,5^). *NLGN1* has been shown to be expressed in endo- and perineural fibroblasts^6^. With respect to immune cells, we could distinguish T cells (*THEMIS*) from macrophages (MΦ: *MRC1*) and dendritic cells (DCs: *CADM1*) (Fig. 1c,d and Supplementary Table 3).

**Extended Data Fig. 3:**
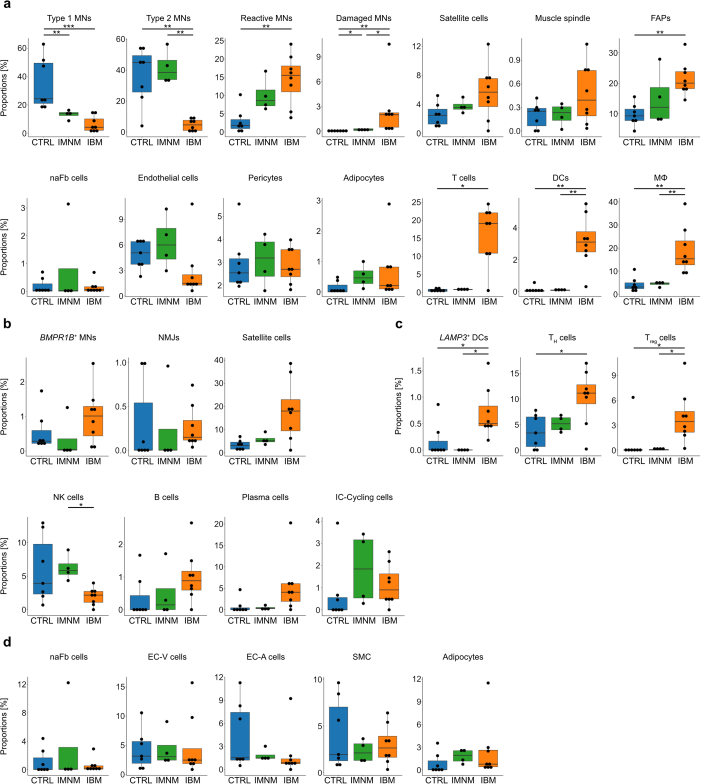
Additional compositional analysis of snRNA-seq clusters. **a** Box plots showing compositional analysis of all identified cell-types within our dataset shown in Fig. 1c-e. Type 1 MN: p(CTRL-IBM) = 9.3 × 10^−4^, p(CTRL-IMNM) = 9.1 × 10^−3^; Type 2 MN: p(CTRL-IBM) = 6.1 × 10^−3^, p(IMNM-IBM) = 6.1 × 10^−3^; Reactive MN: p(CTRL-IBM) = 6.5 × 10^−3^; Damaged MN: p(CTRL-IBM) = 2.4 × 10^−3^, p(CTRL-IMNM) = 1.6 × 10^−2^, p(IMNM-IBM) = 1.6 × 10^−2^; FAPs: p(CTRL-IBM) = 3.7 × 10^−3^; T cells: p(CTRL-IBM) = 1.1 × 10^−2^; DC: p(CTRL-IBM) = 5.9 × 10^−3^, p(IMNM-IBM) = 6.1 × 10^−3^; MΦ: p(CTRL-IBM) = 3.7 × 10^−3^, p(IMNM-IBM) = 6.1 × 10^−3^. **b** Additional box plots showing compositional analysis of myonuclei in CTRL, IMNM, and IBM. Satellite cells: p(CTRL-IBM) = 6.2 × 10^−2^. **c** Additional box plots showing compositional analysis of immune cells in CTRL, IMNM, and IBM. *LAMP3*^*+*^ DC: p(CTRL-IBM) = 2.4 × 10^−2^, p(IMNM-IBM) = 2.2 × 10^−2^; T_H_ cells: p(CTRL-IBM) = 1.8 × 10^−2^; T_reg_ cells: p(CTRL-IBM) = 2.1 × 10^−2^, p(IMNM-IBM) = 2.1 × 10^−2^; NK cells: p(IMNM-IBM) = 1.2 × 10^−2^; Plasma cells: p(CTRL-IBM) = 9.2 × 10^−2^, p(IMNM-IBM) = 9.2 × 10^−2^. **d** Additional box plots showing compositional analysis of endothelial-stromal cells in CTRL, IMNM, and IBM. **a-d** (CTRL, n = 7; IMNM, n = 4; IBM, n = 8); Box plots show median and interquartile range (IQR) of cell/nuclei type proportions, with whiskers extending to the largest and smallest values within 1.5 × IQR range. Two-tailed pairwise Wilcoxon rank-sum tests with Benjamini-Hochberg correction to account for multiple comparisons or Tukey’s HSD tests were performed here between conditions.

With regard to cell type composition, we observed a shift of cell type proportions in both IIM types compared with CTRL muscles, with the strongest changes seen in IBM. We found a relative reduction of type 1 MNs when compared with CTRLs in both IIM types; however, a selective drop-out of type 2 MNs was observed in IBM. This selective type 2 MN loss was paralleled by an increase in reactive and damaged MNs as well as FAP cells, when compared with CTRLs. Furthermore, in IBM we observed an expansion of various immune cell subtypes (MΦ, T cells, DCs) compared with IMNM and CTRL muscles. As expected, the MΦ cluster represented the largest immune cell population in IMNM samples by proportion. Notably, the proportions of naFb cells, endothelial cells, pericytes, adipocytes, satellite cells and muscle spindle cells remained relatively unchanged across all three conditions (Fig. 1e and Extended Data Fig. 3a). To localize individual cell types to spatial coordinates on muscle tissue sections, we mapped cell types based on snRNA-seq to our ST dataset (Fig. 1f). In CTRL muscle biopsies, type 1 and type 2 MN signatures were evenly distributed within tissue sections. In IBM, we observed a loss of type 2 MN signatures in tissue niches enriched for immune cell signatures, including macrophages and T cells as well as reactive and damaged MNs. Conversely, in IBM a loss of type 1 MN signatures appeared to be more diffuse and less restricted to inflamed areas, showing a similar pattern to that seen in IMNM tissue sections.

In summary, we found marked reductions of both type 1 and 2 MNs in IBM with a selective and spatially restricted loss of type 2 MN signatures in inflamed tissue niches in IBM.

### Increase in damaged MNs mirrors type 2 loss in IBM

**Fig. 2:**
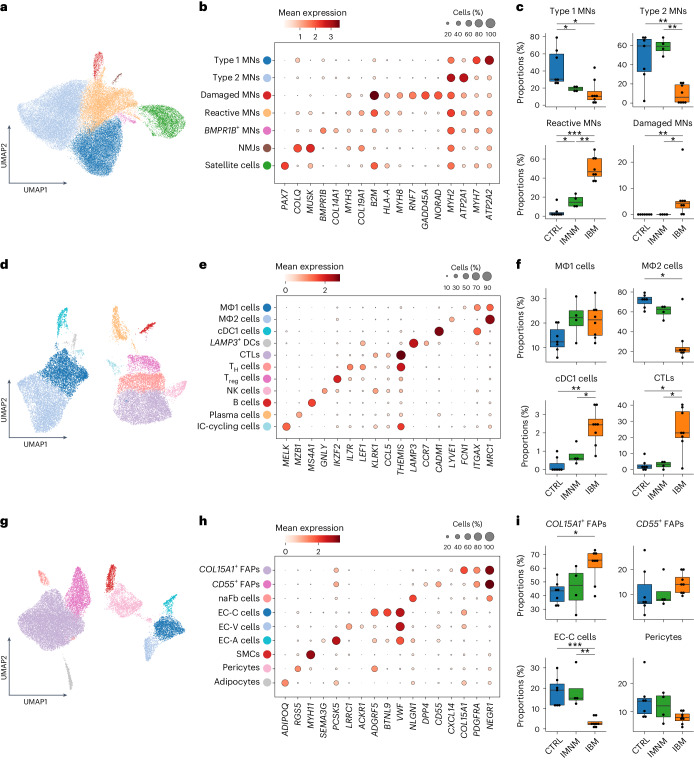
Subclustering of MNs, immune, endothelial and stromal cells. **a**, UMAP visualization showing all MNs (*n* = 49,633) present in the snRNA-seq dataset from all conditions. **b**, Dot plot showing marker genes of MN clusters. **c**, Compositional analysis of type 1 MNs (*P*(CTRL-IBM) = 2.1 × 10^−2^; *P*(CTRL-IMNM) = 1.8 × 10^−2^), type 2 MNs (*P*(CTRL-IBM) = 6.1 × 10^−3^; *P*(IMNM-IBM) = 6.1 × 10^−3^), reactive MNs (*P*(CTRL-IBM) = 9.3 × 10^−4^; *P*(CTRL-IMNM) = 2.4 × 10^−2^; *P*(IMNM-IBM) = 6.1 × 10^−3^) and damaged MNs (*P*(CTRL-IBM) = 5.2 × 10^−3^; *P*(IMNM-IBM) = 1.7 × 10^−2^) in CTRL, IMNM and IBM (CTRL, *n* = 7; IMNM, *n* = 4; IBM, *n* = 8). **d**, UMAP visualization showing all immune cells (*n* = 20,620) present in the snRNA-seq dataset from all conditions. **e**, Dot plot showing marker genes of immune cell clusters. **f**, Compositional analysis of MΦ1 cells (*P*(CTRL-IBM) = 1.0 × 10^−1^, *P*(CTRL-IMNM) = 1.4 × 10^−1^), MΦ2 cells (*P*(CTRL-IBM) = 1.1 × 10^−2^), cDC1 cells (*P*(CTRL-IBM) = 5.4 × 10^−3^; *P*(IMNM-IBM) = 2.4 × 10^−2^) and CTLs (*P*(CTRL-IBM) = 2.7 × 10^−2^; *P*(IMNM-IBM) = 4.2 × 10^−2^) in CTRLs, IMNM and IBM (CTRLs, *n* = 7; IMNM, *n* = 4; IBM, *n* = 8). **g**, UMAP visualization showing all endothelial–stromal cells (*n* = 22,781) present in the snRNA-seq dataset from all conditions. **h**, Dot plot showing marker genes of identified endothelial–stromal cells. **i**, Compositional analysis of *COL15A1*-expressing FAPs (*P*(CTRL-IBM) = 2.8 × 10^−2^), *CD55*-expressing FAPs, EC-C cells (*P*(CTRL-IBM) = 9.3 × 10^−4^; *P*(IMNM-IBM) = 6.1 × 10^−3^) and pericytes in CTRL, IMNM and IBM (CTRL, *n* = 7; IMNM, *n* = 4; IBM, *n* = 8). Box plots in **c**, **f** and **i** show the median and interquartile range (IQR) of cell/nuclei-type proportions, with whiskers extending to the largest and smallest values within 1.5× the IQR range. Two-tailed, pairwise Wilcoxon’s rank-sum tests with Benjamini–Hochberg correction to account for multiple comparisons or Tukey’s HSD test were performed between conditions in **c**, **f** and **i**. ^*^P < 0.05, ^**^P < 0.01, ^***^P < 0.001.

Next, we performed subclustering and focused on MN subtypes characterized by subtype marker gene expression: type 1 MNs (*MYH7*, *ATP2A2*), type 2 MNs (*MYH2*, *ATP2A1*), damaged MNs (*RNF7*, *GADD45A*, *NORAD*), reactive MNs (*MYH3*, *COL19A1*, *MYH8*)^7^, satellite cells (*PAX7*) and, more specifically, MNs related to neuromuscular junctions (NMJs; *MUSK* and *COLQ*) and *BMPR1B*-expressing MNs (*BMPR1B* and *COL14A1*) (Fig. 2a,b). Of note, *GADD45A* expression was described in the context of DNA damage and muscle atrophy, in particular during fasting and denervation^8–10^. *NORAD* is a lncRNA that regulates messenger RNA translation by inhibiting Pumilio proteins (PUM1 and PUM2) known to prevent mRNA transcripts from translation^11,12^. *RNF7* encodes for Ring finger protein 7 (RNF7), also called sensitive to apoptosis gene (SAG), a known part of the SCF (SKP1-CUL-F-box proteins) E3 ubiquitin ligase and known for its antioxidative function when acting alone^13^. Reactive MNs lacked expression of genes associated with atrophy (for example, *GADD45A*) but showed expression of embryonic and fetal myosin heavy chains (*MYH3* and *MYH8*). Furthermore, both reactive and damaged MN subtypes were characterized by expression of genes associated with inflammation and antigen presentation (for example, *B2M* and *HLA-A*) (Fig. 2b and Supplementary Table 4). *BMPR1B*-expressing MNs represented only a small population of MNs, which might be associated with hypertrophic or denervated myofibers^14,15^.

**Extended Data Fig. 4:**
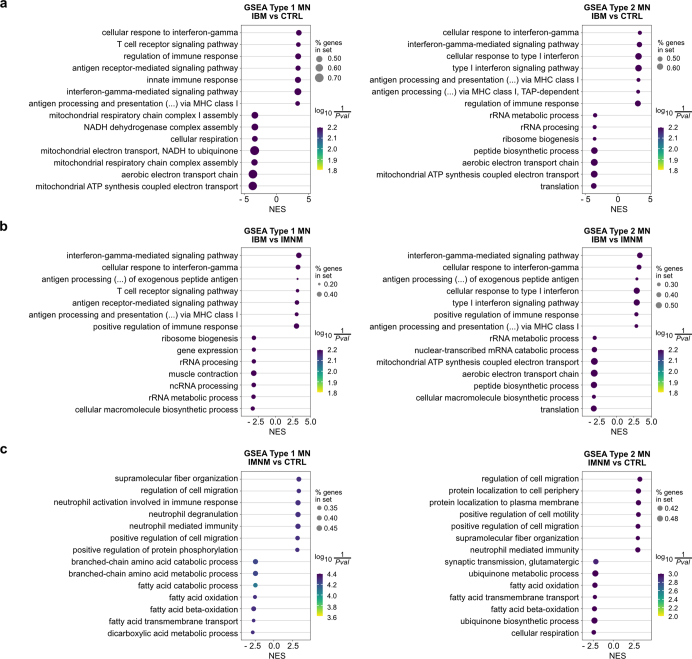
Gene set enrichment analysis (GSEA) of Type 1 and 2 MN between CTRL and IIM samples. **a** Dot plots showing the top 7 enriched gene sets based on the normalized enrichment score (NES) in Type 1 and 2 MN comparing IBM with CTRL muscle. **b** Dot plots showing the top 7 enriched gene sets based on the NES in Type 1 and 2 MN comparing IBM with IMNM muscle. **c** Dot plots showing the top 7 enriched gene sets based on the NES in Type 1 and 2 MN comparing IMNM with CTRL muscle. % genes in set indicates the proportion of enriched genes within a gene set. The ‘signal_to_noise’ - method was used as statitistical test for data analysis.

Compositional analysis of MN subtypes revealed marked differences within the proportions of type 1 MNs, type 2 MNs, reactive MNs, damaged MNs and satellite cells across IBM, IMNM and CTRLs. Specifically, we confirmed a loss of type 1 MNs in both IBM and IMNM relative to CTRLs and a selective loss of type 2 MNs in IBM relative to CTRL and IMNM muscles. Reactive MNs exhibited a disease-specific increase in both IMNM and IBM with a stronger increase in IBM compared with CTRLs and IMNM. Damaged MNs, however, were exclusively found in IBM based on subcluster analysis. Furthermore, we observed a trend of higher proportions of satellite cells in IBM compared with CTRLs. Notably, we detected no differences of NMJs and *BMPR1B*-expressing MN populations (Fig. 2c and Extended Data Fig. 3b). To further investigate dysregulated signaling pathways in IIM, we performed gene set enrichment analysis (GSEA) in type 1 and type 2 MNs. In particular, we found inflammatory gene sets to be enriched in type 1 and 2 MNs in IBM relative to CTRLs and IMNM (for example, interferon-γ-mediated signaling pathway, gene ontology (GO) 0060333). Conversely, mitochondrial pathways (for example, mitochondrial respiratory chain complex I assembly, GO 0032981) were downregulated in type 1 MNs compared with CTRLs. Furthermore, we noted gene sets involved in lipid catabolism (for example, fatty acid β-oxidation, GO 0006635) to be downregulated in IMNM type 1 and 2 MNs versus CTRLs (Extended Data Fig. 4 and Supplementary Tables 7–12).

Collectively, we found IIM-specific reactive MNs to be more abundant in both types of IIM, and damaged MNs to be selectively expanded in IBM. Enrichment of reactive and damaged MNs was paralleled by a loss of type 1 MNs in both IMNM and IBM and an IBM-specific loss of type 2 MNs.

### T cell expansion and loss of homeostatic macrophages in IBM

Immune cell subcluster analysis revealed two distinct macrophage populations: MΦ1 (*FCN1*, *ITGAX* and *MRC1*)^16,17^ and MΦ2 (*LYVE1* and *MRC1*)^18^ with MΦ1 cells resembling a proinflammatory subtype and MΦ2 cells featuring homeostatic macrophages based on gene expression. In addition, we identified two distinct populations of DCs: conventional type 1 DCs (cDC1 cells: *CADM1* and *ITGAX*) and *LAMP3*-expressing DCs (*LAMP3* and *CCR7*), as well as three distinct T cell populations: cytotoxic T lymphocytes (CTLs: *KLRK1*, *CCL5* and *THEMIS*), helper T cells (T_H_ cells: *LEF1*, *IL7R* and *THEMIS*), regulatory T cells (T_reg_ cells: *IKZF2* and *THEMIS*) and one natural killer (NK) cell population (*GNLY* and *KLRK1*). Furthermore, we could distinguish B cells (*MS4A1*) from plasma cells (*MZB1*) and found one subtype of proliferating immune cells (IC-cycling cells: *MELK*) (Fig. 2d,e and Supplementary Table 5).

Compositional analysis revealed an IIM-specific trend toward higher proportions of MΦ1 cells in both IIM types versus CTRLs, whereas MΦ2 cells were selectively reduced in IBM muscles (Fig. 2f). With regard to DCs, cDC1 cells and *LAMP3*-expressing DCs were strongly increased in IBM muscles relative to CTRLs and IMNM. Proportions of CTLs and T_reg_ cells were higher in IBM compared with CTRLs and IMNM, whereas T_H_ cells were higher in only IBM compared with CTRLs, but not IMNM. Of note, we found that NK cells were increased in IMNM relative to IBM. We noted a trend of higher proportions of plasma cells in IBM compared with CTRLs and IMNM, but no differences were seen for B cells and proliferating immune cells (Fig. 2f and Extended Data Fig. 3c).

To summarize, subcluster analysis identified a selective reduction of homeostatic macrophages and increased proportions of CTLs and cDC1 cells in IBM.

### Shifts in fibroblast and endothelial cell ratios in IBM

To gain more insight into endothelial and stromal cells, we performed a subcluster analysis and identified three FAP/fibroblast (Fb) subtypes based on gene expression (Fig. 2g,h, Extended Data Fig. 3d and Supplementary Table 6): the FAP cluster (Fig. 1c–d) separated into two distinct subclusters of *COL15A1*-expressing FAPs (*COL15A1*, *CXCL14*, *PDGFRA* and *NEGR1*) and *CD55*-expressing FAPs (*CD55*, *DPP4*, *PDGFRA* and *NEGR1*). Subtypes of FAPs/Fbs with similar expression signatures have been described before in both human and mouse skeletal muscle tissue studies^19–22^. One study suggested that *COL15A1*-expressing FAPs represented endomysial Fbs and FAPs corresponding to our *CD55*-expressing FAPs represent perimysial Fbs^21^. Our subcluster analysis revealed no further separations of the naFb cell (*NLGN1* and *NEGR1*) population (Fig. 1d). Moreover, we could distinguish three subtypes of endothelial cells: capillary endothelial cells (EC-C cells: *ADGRF5*, *BTNL9* and *VWF*), venous endothelial cells (EC-V cells: *LRRC1*, *ACKR1* and *VWF*) and arterial endothelial cells (EC-A cells: *SEMA3G*, *PCSK5* and *VWF*)^23^. Finally, we could also identify smooth muscle cells (SMCs: *MYH11*), pericytes (*RGS5*) and adipocytes (*ADIPOQ*).

By compositional analysis, we found that *COL15A1*-expressing FAPs were more abundant in IBM than in CTRL muscles. Furthermore, we noted a strong loss of EC-C cells in IBM compared with CTRL and IMNM muscles. However, we did not observe differences in proportions between the different entities in other endothelial–stromal cell subtypes (Fig. 2i and Extended Data Fig. 3d).

Collectively, we could identify an increase in *COL15A1*-expressing FAPs and a decrease in capillary endothelial cells in IBM.

### IBM-specific cell state expansion of endomysial FAPs

**Fig. 3:**
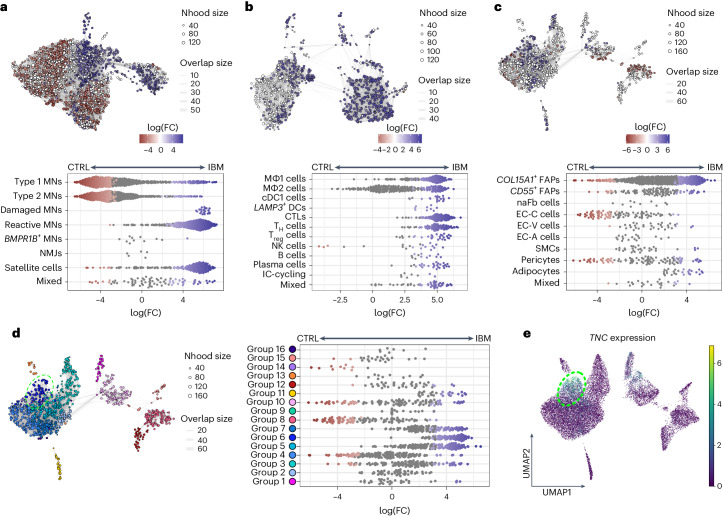
Differential abundance analysis between CTRLs and IBM. **a**, Top, neighborhood graph visualizing the results of differential abundance analysis between CTRL and IBM MNs. Bottom, beeswarm plot showing the log(fold change) (log(FC)) of abundance in neighborhoods between CTRL and IBM MNs. **b**, Top, neighborhood graph visualizing the results of differential abundance analysis between CTRLs and IBM in immune cells. Bottom, beeswarm plot showing the log(FC) of abundance in neighborhoods between CTRLs and IBM in immune cells. **c**, Top, neighborhood graph visualizing the results of differential abundance analysis between CTRLs and IBM in endothelial–stromal cells. Bottom, beeswarm plot showing the log(FC) of abundance in neighborhoods between CTRLs and IBM in endothelial–stromal cells. **d**, Left, neighborhood plot showing the grouping of neighborhoods as shown in **c** based on the number of shared cells and direction of fold-changes of abundance between CTRLs and IBM in endothelial–stromal cells. Right, beeswarm plot showing log(FC) of abundances of neighborhoods within neighborhood groups in endothelial–stromal cells. **e**, UMAP visualization showing *TNC* expression within endothelial–stromal cell subpopulations in CTRL, IMNM and IBM muscle. Note, contours in **d** and **e** highlight neighborhoods/cells of group 6. For neighborhood graphs in **a**–**d**: every node represents a neighborhood, colored by log(FC) between CTRLs (red) and IBM (blue). Neighborhoods with no detected differential abundance (corrected spatial FDR > 0.1) are colored white (**a**–**c**). The node size correlates with the number of nuclei within a neighborhood (Nhood size) and the graph edge width indicates the number of overlapping cells between adjacent neighborhoods (overlap size). The position of nodes is based on the position of the nuclei shown in the UMAP visualizations in Fig. 2a,d,g. For beeswarm plots in **a**–**d**: every dot represents a neighborhood, colored by log(FC) between CTRLs (red) and IBM (blue). Neighborhoods with no detected differential abundance (corrected spatial FDR > 0.1) are colored gray. Note, ‘mixed’ indicates that <70% of nuclei within a neighborhood originate from one single cluster.

**Extended Data Fig. 5:**
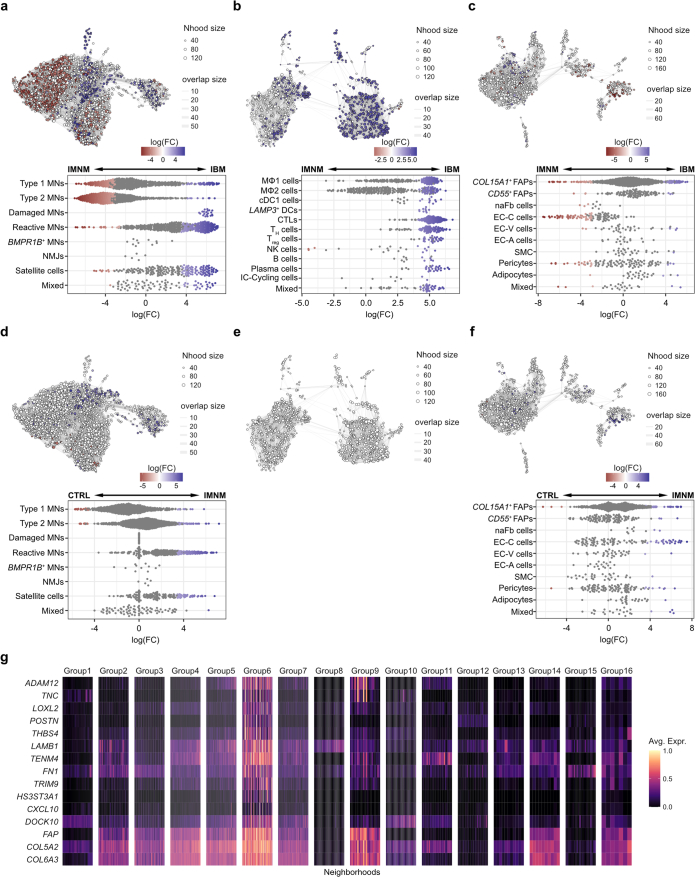
Differential abundance analysis between IMNM versus IBM and CTRL versus IMNM. **a** Top: Neighborhood graph visualizing results of differential abundance analysis (DAA) between IMNM and IBM in myonuclei. Bottom: Beeswarm plot showing log(fold-changes) (log(FC)) of abundances in neighborhoods between IMNM and IBM in myonuclei. **b** Top: Neighborhood graph visualizing results of DAA between IMNM and IBM in immune cells. Bottom: Beeswarm plot showing log(FC) of abundances in neighborhoods between IMNM and IBM in immune cells. **c** Top: Neighborhood graph visualizing results of DAA between IMNM and IBM in endothelial-stromal cells. Bottom: Beeswarm plot showing log(FC) of abundances in neighborhoods between IMNM and IBM in endothelial-stromal cells. **d** Top: Neighborhood graph visualizing results of DAA between CTRL and IMNM in myonuclei. Bottom: Beeswarm plot showing log(FC) of abundances in neighborhoods between CTRL and IMNM in myonuclei. **e** Neighborhood graph visualizing results of DAA between CTRL and IMNM in immune cells. **f** Top: Neighborhood graph visualizing results of DAA between CTRL and IMNM in endothelial-stromal cells. Bottom: Beeswarm plot showing log(FC) of abundances in neighborhoods between CTRL and IMNM in endothelial-stromal cells. **g** Heatmap visualizing average expression of selected marker genes of Group6. For neighborhood graphs in **a** - **f**: Nodes represent neighborhoods, colored by log(FC) between IMNM (red) and IBM (blue) (**a** – **c**) or CTRL (red) and IMNM (blue) (**d** - **f**). Neighborhoods with no detected differential abundance (corrected spatialFDR > 0.1) are colored white (**a** - **f**). Node size correlates to the numbers of nuclei within a neighborhood (Nhood size) and graph edge width indicates the number of overlapping cells between adjacent neighborhoods (overlap size). Node positions are based on positions of nuclei as shown in Fig. 2a, d, g. For beeswarm plots in **a – d**, and **f**: Every dot represents a neighborhood, colored by log(FC) between IMNM (red) and IBM (blue) (**a** – **c**) or CTRL (red) and IMNM (blue) (**d** and **f**). Neighborhoods with no detected differential abundance (corrected spatialFDR > 0.1) are colored grey. ‘Mixed’ indicates < 70% of nuclei within a neighborhood originate from one cluster.

To gain a deeper insight into possible compositional changes within cell types, a differential abundance analysis was performed. Using an unsupervised workflow, nuclei were assigned to partially overlapping neighborhoods^24^ to validate results observed in the compositional analysis above (Fig. 2c,f,i and Extended Data Fig. 3b–d). For MNs, we confirmed a loss of type 1 and more pronounced type 2 MNs in IBM. Furthermore, we observed higher abundance of damaged MNs, reactive MNs and satellite cells in IBM compared with CTRL and IMNM muscle tissues (Fig. 3a and Extended Data Fig. 5a). The strongest changes in MN abundance between CTRLs and IMNMs were found in reactive MNs and satellite cells, with both being more abundant in IMNM (Extended Data Fig. 5d). With regard to immune cells, we observed higher abundance of all identified immune cell types in IBM. Homeostatic MΦ2 cells remained the most abundant immune cell subtype in CTRLs and IMNM (Fig. 3b and Extended Data Fig. 5b), confirming previous compositional analysis results (Fig. 2f). Of note, we did not observe different abundances in immune cells between CTRLs and IMNM (Extended Data Fig. 5e). With respect to endothelial and stromal cells, we found higher abundances of *COL15A1*-expressing FAPs and a loss of EC-C cells in IBM (Fig. 3c and Extended Data Fig. 5c), confirming the compositional analysis results above. The strongest changes in endothelial–stromal cell abundances between CTRLs and IMNM were found in EC-C cells and pericytes, with both cell types being more abundant in IMNM muscle (Extended Data Fig. 5f). Next, we focused on endothelial–stromal cells and subdivided neighborhoods into different groups based on the number of shared cells and the direction of fold-changes between CTRLs and IBM. Accordingly, in IBM we found a highly abundant neighborhood group (group 6) within the *COL15A1*-expressing FAP population that was characterized by expression of genes associated with fibrosis, tissue remodeling (for example *TNC*, *POSTN* and *LOXL2*)^25–29^ and inflammation (for example, *CXCL10*) representing an IBM-specific cell state of endomysial FAPs (Fig. 3c–e, Extended Data Fig. 5c,f,g and Supplementary Table 13).

Taken together, differential abundance analysis helped validate patterns of cell type compositions and characterize an IBM-specific cell state of endomysial *COL15A1*-expressing FAPs.

### Spatial transcriptomics reveals muscle niche compositions

**Fig. 4:**
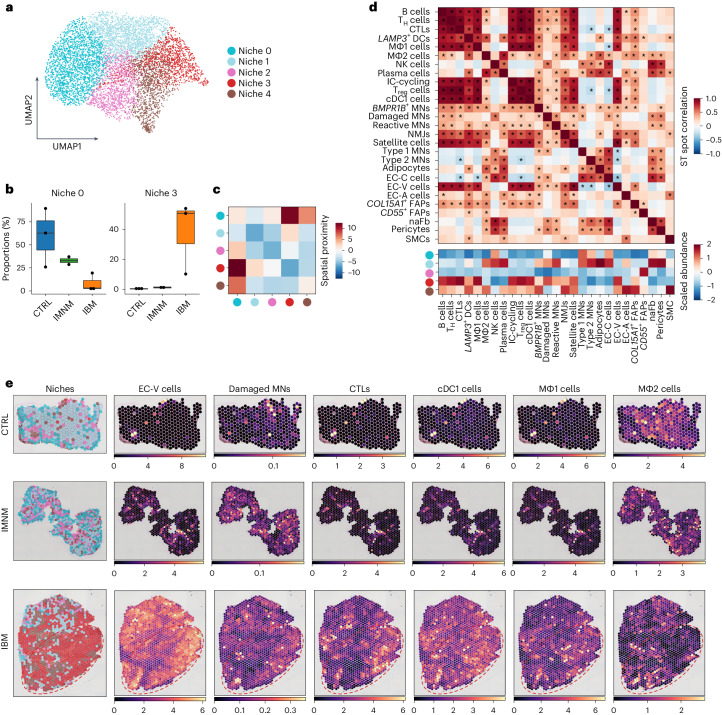
ST reveals IBM-specific inflammatory tissue niche. **a**, UMAP visualization showing tissue niches of CTRL, IMNM and IBM muscle (spots, *n* = 7,462). **b**, Compositional analysis of tissue niches in CTRLs, IMNM and IBM (CTRLs, *n* = 3; IMNM, *n* = 2; IBM, *n* = 3) showing reduced proportions of myofiber-associated niche 0 in IBM compared with CTRLs and increased proportions of immune cell-associated niche 3 in IBM compared with CTRLs. Box plots show median and IQR of tissue niche proportions, with whiskers extending to the largest and smallest values within the 1.5× IQR. **c**, Heatmap showing spatial proximity of tissue niches. **d**, Heatmaps showing the correlation of spatial locations of cell types identified by snRNA-seq (upper heatmap) and scaled abundance of these cell types within tissue niches. |*r*|: ^*^P > 0.25. **e**, Tissue niches shown on tissue sections of CTRL, IMNM and IBM muscle in the left column. The right columns show locations of selected cell types identified in snRNA-seq based on deconvolution of ST data (CTRL, *n* = 3; IMNM, *n* = 2; IBM, *n* = 3). Note, the red contours highlight areas with a low abundance of type II MNs in IBM (Fig. 1f).

**Extended Data Fig. 6:**
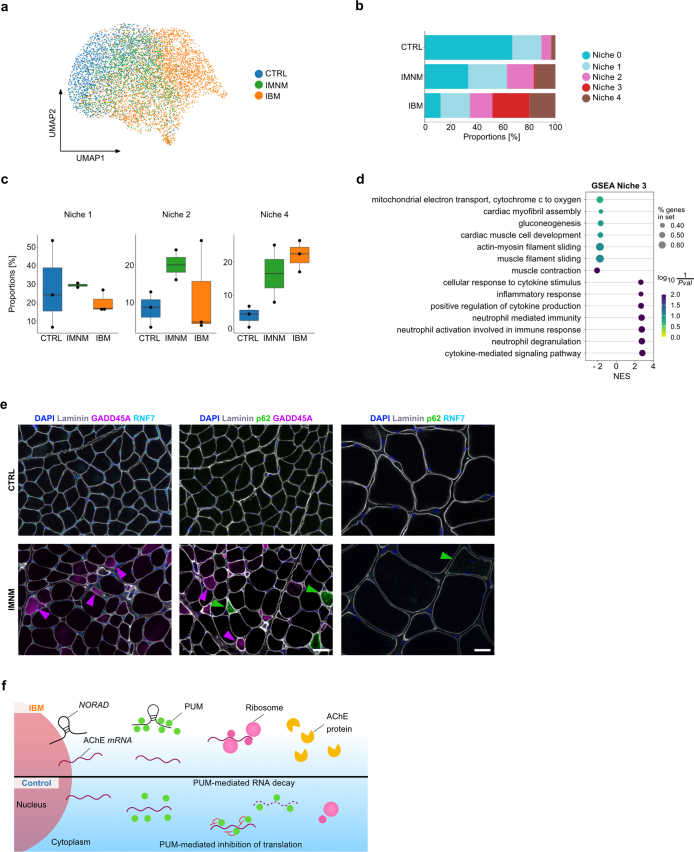
Additional analysis of ST and IHC results between CTRL and IIM samples. **a** UMAP visualization showing ST spots colored by condition. **b** Bar plot showing compositional analysis of tissue niches in CTRL, IMNM and IBM muscle. **c** Box plots showing compositional analysis of tissue niches in CTRL (n = 3), IMNM (n = 2), and IBM (n = 3). Box plots show median and interquartile range (IQR) of tissue niche proportions, with whiskers extending to the largest and smallest values within 1.5 x IQR range. **d** Dot plot showing the top 7 up- and downregulated gene sets based on the NES in Niche 3 compared to all the other niches. % genes in set indicates the proportion of enriched genes within a gene set. The ‘signal_to_noise’ - method was used as statitistical test for data analysis. **e** Immunohistochemistry (IHC) showing expression of Laminin, p62, GADD45A, and RNF7 in CTRL and IMNM muscle on the protein level. Note purple arrowheads pointing at GADD45A^+^ myofibers, and green arrowheads pointing at p62^+^ myofibers. Scale bar (left and middle image) = 50 μm, scale bar (right image) = 20 μm. **f** Sketch showing links between genomic stress and functional denervation in IBM.

Spatially resolved gene expression revealed five distinct niches corresponding to specific biological functions that were selectively enriched in IIM and CTRL muscles (Fig. 4a and Extended Data Fig. 6a,b). Two niches resembled myofibers (niches 0 and 1), one niche connective/fibrotic tissue (niche 2), one niche inflamed tissue (niche 3) and one niche blood vessels and perivascular connective tissue (niche 4) (Supplementary Table 14). Niche 0 appeared to be most prevalent in CTRL and IMNM muscles, niches 1 and 2 showed similar proportions across all three conditions, whereas niches 3 and 4 were expanded in IBM compared with CTRL muscles (Fig. 4a,b and Extended Data Fig. 6c). In addition, we performed GSEA on genes enriched in niche 3 compared with the other tissue niches. In niche 3, we found an upregulation of gene sets associated with inflammation (for example, cytokine-mediated signaling pathway, GO: 0019221) and a downregulation of gene sets associated with myofiber function (for example, muscle contraction, GO: 0006936) versus other tissue niches (Extended Data Fig. 6d and Supplementary Table 15). To investigate the spatial relationship between the identified tissue niches, we performed a neighborhood enrichment analysis and found a strong spatial proximity between the homeostatic myofiber niche 0 and the IBM-specific niche 3, as well as between niche 0 and niche 4 (Fig. 4c). Mapping of cell types to spatial coordinates enabled us to localize cell types and define the spatial niche composition based on cell type marker expression (Fig. 4d,e). Both niches 0 and 1 contained mainly type 1 MNs and type 2 MNs, whereas niche 1 also contained signatures related to damaged MNs, reactive MNs, homeostatic macrophages (MΦ2 cells), NK cells, plasma cells, adipocytes, EC-C subtypes, pericytes and naFb cells, overall suggesting changes related to tissue inflammation and remodeling. Niche 2 represented a second tissue-remodeling niche characterized by gene expression related to *CD55*-expressing FAPs and, probably, perimysial tissue. Niche 3 was mainly formed by immune cells including CTLs, T_H_ cells, T_reg_ cells, MΦ1 cells, cDC1 cells, B cells, IC-cycling cells as well as EC-V cells, hence resembling a tissue niche related to early inflammatory changes. Notably, damaged and reactive MNs were also present in niche 3. Niche 4 represented a second immune-related tissue compartment that appeared less inflamed than niche 3, with, however, evidence for tissue remodeling enriched for *LAMP3*-expressing DCs and MΦ2 cells as well as EC-A cells and expression related to *COL15A1*-expressing FAPs.

Furthermore, correlation analysis revealed two distinct spatial patterns of immune cells (Fig. 4d,e). For example, T cell subtypes, B cells, cDC1 cells and proinflammatory macrophages (MΦ1 cells) appeared to be associated with EC-V cells, reactive MNs, damaged MNs and NMJs, suggesting acute myofiber damage. Tissue-resident/homeostatic macrophages (MΦ2 cells), conversely, were spatially more closely related to *LAMP3*-expressing DCs, plasma cells, type 2 MNs and adipocytes, suggesting a less active and, potentially, more chronic inflammatory tissue state. Focusing on EC subtypes, we found the largest differences between EC-C and EC-V subtypes. EC-C cells appeared to be negatively spatially correlated with CTLs, T_reg_ cells, MΦ1 cells and EC-V subtypes. Conversely, the presence of EC-V cells was positively correlated with other immune cell types (excluding MΦ2 cells, NK cells and plasma cells), *BMPR1B*-expressing MNs, reactive and damaged MNs, NMJs, satellite cells and *COL15A1*-expressing FAPs. Also, we noted that *COL15A1*-expressing FAPs were spatially linked to immune cells, EC-V cells, satellite cells, NMJs and *CD55*-expressing FAPs. In contrast, naFb cells appeared to be closely associated with type 1 and type 2 MNs, damaged MNs, reactive MNs, EC-C cells, adipocytes, pericytes, MΦ2 cells, NK cells and plasma cells (Fig. 4d,e).

In summary, we could identify various spatially restricted tissue niches present in CTRLs and IIM with various muscle damage and immune cell subtype gene expression signatures being spatially linked and enriched in IBM-specific tissue niches.

### Type 2A myofiber vulnerability linked to genomic stress

**Fig. 5:**
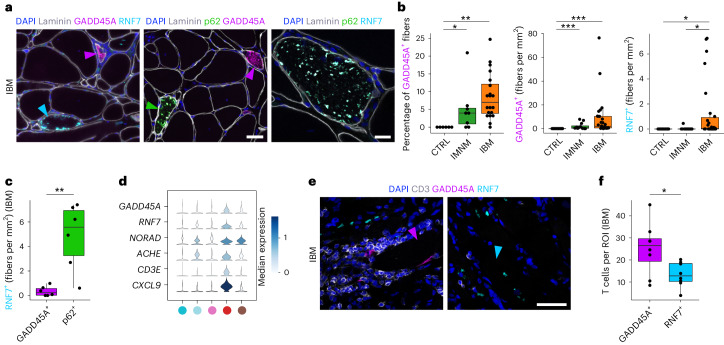
*GADD45A*-associated myofiber infiltration of T cells in IBM. **a**, IHC for laminin, p62, GADD45A and RNF7 in IBM. Note the protein aggregates containing p62 and RNF7 in a myofiber in the right image, and purple arrowheads pointing at GADD45A^+^ myofibers, a cyan arrowhead at a RNF7^+^ myofiber and a green arrowhead at a p62^+^ myofiber. Scale bar, 50 μm (left and middle image), 20 μm (right image). **b**, Box plots showing quantification of GADD45A^+^ myofibers and RNF7^+^ myofibers in CTRLs, IMNM and IBM (left plot: CTRLs, *n* = 6; IMNM, *n* = 8; IBM, *n* = 20, *P*(CTRL-IBM) = 1.5 × 10^−3^, *P*(CTRL-IMNM) = 1.9 × 10^−2^; middle plot: CTRLs, *n* = 12; IMNM, *n* = 12; IBM, *n* = 28, p(CTRL-IBM) = 3.7 × 10^−6^, *P*(CTRL-IMNM) = 1.9 × 10^−4^; right plot: CTRLs, *n* = 11; IMNM, *n* = 12; IBM, *n* = 26, *P*(CTRL-IBM) = 1.7 × 10^−2^, *P*(IMNM-IBM) = 2.3 × 10^−2^). **c**, Box plot showing quantification of RNF7^+^ myofibers coexpressing GADD45A or p62 in IBM (*n* = 6, *P* = 9.1 × 10^−3^). **d**, Stacked violin plots showing expression of the damaged MN markers *GADD45A*, *RNF7* and *NORAD* and *ACHE* and inflammation markers *CD3E* and *CXCL9* within tissue niches. **e**, IHC for GADD45A^+^ and RNF7^+^ myofibers to CD3^+^ T cells in IBM muscle with T cells invading a GADD45A^+^ myofiber. A purple arrowhead points at a GADD45A^+^ myofiber and a cyan arrowhead at a RNF7^+^ myofiber. Scale bar, 50 μm. **f**, Box plot showing the amount of CD3^+^ T cells around GADD45A^+^ or RNF7^+^ myofibers per region of interest (ROI) per IBM sample (*n* = 8, *P* = 2.2 × 10^−2^). Box plots in **b**, **c** and **f** show the median and IQR, with whiskers extending to the largest and smallest values within the 1.5× IQR range. Two-tailed, pairwise Wilcoxon’s rank-sum tests with Benjamini–Hochberg correction to account for multiple comparisons were performed between conditions in **b**. Two-tailed, paired Student’s *t*-tests were performed between groups in **c** and **f**. ^*^P < 0.05, ^**^P < 0.01, ^***^P < 0.001.

Next, we focused on the IBM-specific damaged MN subtype characterized by expression of *RNF7, GADD45A* and *NORAD* (Figs. 1d and 2b). Based on ST analysis, damaged MN signatures were spatially associated with inflammatory gene signatures (for example, *CD3E* and *CXCL9*) in niche 3 (Figs. 4d and 5d). To validate these findings, we performed immunohistochemistry (IHC) experiments and confirmed that GADD45A and RNF7 proteins accumulated in IBM relative to CTRL muscles with, however, GADD45A^+^ fibers being more strongly associated with CD3^+^ T cell infiltrates than RNF7 in IBM. Of note, RNF7 upregulation was specific to IBM, but IMNM muscles also showed a higher presence of GADD45A proteins in contrast to CTRLs. We also investigated proportions of GADD45A^+^ myofibers and found a similar pattern with a relative upregulation in IBM and IMNMs relative to CTRLs (Fig. 5a,b,e,f and Extended Data Fig. 6e). Although both marker genes (*GADD45A* and *RNF7*) were enriched in the damaged MN subtype, we noted a spatial segregation of myofibers upregulating either GADD45A or RNF7 in IBM. Besides the association with T cells, we explored the relationship of RNF7 with p62 protein expression, because both are involved in protein degradation within cells, and p62^+^ aggregates are a common histopathological finding in IBM^30^. Based on IHC, we found an upregulation of RNF7 in myofibers associated with p62 protein aggregates (but not GADD45A) (Fig. 5a,c), suggesting two spatially and functionally distinct damage pathways in IBM muscles linked to either cell/genomic stress and denervation (GADD45A) or protein degradation/autophagy (p62/RNF7).

**Fig. 6:**
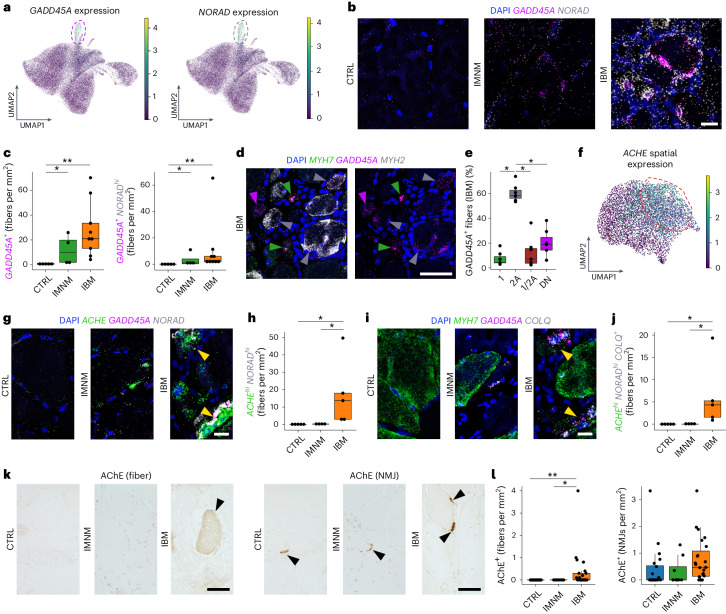
Elevated ACHE expression in IBM is associated with lncRNA *NORAD* upregulation. **a**, UMAP visualization showing *GADD45A* and *NORAD* expression within MNs in CTRLs, IMNM and IBM. **b**, SmFISH for *GADD45A* and *NORAD* in CTRLs, IMNM and IBM. **c**, Quantification of *GADD45A*^*+*^ (*P*(CTRL-IBM) = 3.0 × 10^−3^, *P*(CTRL-IMNM) = 2.4 × 10^−2^) and *GADD45A*^*+*^ *NORAD*^hi^ myofibers (*P*(CTRL-IBM) = 9.0 × 10^−3^, *P*(CTRL-IMNM) = 2.3 × 10^−2^) based on smFISH in CTRLs (*n* = 5), IMNM (*n* = 4) and IBM (*n* = 9). **d**, SmFISH for *GADD45A* and *MYH7* (type 1) and *MYH2* (type 2 A) in IBM. The arrowheads indicate *GADD45A*^*+*^ type 1 (green), *GADD45A*^*+*^ type 2A (gray) and *MYH7*^*−*^*MYH2*^*−*^*GADD45A*^*+*^ (purple) myofibers. **e**, Proportions of *GADD45A* expression in different myofiber types in IBM (*n* = 7) (*P*(2A-1) = 3.1 × 10^−2^, *P*(2A-1/2 A) = 3.1 × 10^−2^, *P*(2A-DN) = 3.1 × 10^−2^). **f**, UMAP visualization showing spatial *ACHE* expression in CTRLs, IMNM and IBM. **g**, SmFISH showing expression of *GADD45A*, *NORAD* and *ACHE* in CTRLs, IMNM and IBM. The yellow arrowheads point at *ACHE*^*hi*^*NORAD*^*hi*^ myofibers. **h**, Quantification of *ACHE*^*hi*^*NORAD*^*hi*^ myofibers in CTRLs (*n* = 5), IMNM (*n* = 4) and IBM (*n* = 5) (*P*(CTRL-IBM) = 2.4 × 10^−2^, *P*(IMNM-IBM) = 2.4 × 10^−2^). **i**, SmFISH for *GADD45A*, *MYH7* and *COLQ* in CTRLs, IMNM and IBMs. The yellow arrowheads indicate *COLQ*^*+*^ myofibers, coexpressing *ACHE* and *NORAD* (see **g**). **j**, *ACHE*^*hi*^*NORAD*^*hi*^*COLQ*^*+*^ myofiber quantification in CTRLs (*n* = 5), IMNM (*n* = 4) and IBM muscles (*n* = 5) (*P*(CTRL-IBM) = 2.2 × 10^−2^, *P*(IMNM-IBM) = 2.7 × 10^−2^). **k**, IHC for AChE^+^ myofibers and NMJs in IBMs. The black arrowheads point at AChE^+^ myofibers (left) and NMJs (right), respectively. Scale bar, 50 μm. **l**, Quantification of AChE^+^ myofibers and NMJs in CTRLs (*n* = 20), IMNM (*n* = 8) and IBM (*n* = 23) (left: *P*(CTRL-IBM) = 2.1 × 10^−3^, *P*(IMNM-IBM) = 3.2 × 10^−2^; right: *P*(CTRL-IBM) = 7.6 × 10^−2^). Contours in **a** and **f** highlight areas with high expression of displayed genes. Box plots in **c**, **e**, **h**, **j** and **l** show the median and IQR, with whiskers extending to the largest and smallest values within 1.5× the IQR. Here, two-tailed, pairwise Wilcoxon’s rank-sum (**c**, **h**, **j** and **l**) or signed-rank (**e**) tests with Benjamini–Hochberg correction to account for multiple comparisons were performed. ^*^P < 0.05, ^**^P < 0.01. Scale bars, 20 µm (**b**, **g** and **i**), 50 µm (**d** and **k**).

Next, based on snRNA-seq, we confirmed that both stress marker genes *GADD45A* and *NORAD* were specifically enriched in the damaged MN cluster (Figs. 2b and 6a). This pattern was validated by ST, where *GADD45A* was upregulated in the IBM-specific tissue niche 3 (Fig. 5d). By multiplex single molecule (sm)FISH, we could demonstrate that *GADD45A* and *GADD45A*/*NORAD*^*hi*^-coexpressing myofibers were upregulated in IIM muscles with the highest density in IBM (Fig. 6b,c). We then addressed whether *GADD45A* upregulation was specific to a certain myofiber subtype considering that type 2 MNs were selectively lost in IBM muscles. To address myofiber vulnerability, we conducted a multiplex smFISH analysis investigating myofiber gene expression for *GADD45A* with *MYH7* and *MYH2*, encoding for type 1- and type 2A-specific myosin heavy chains, respectively. Notably, besides type 1 and 2A myofibers, we found myofibers expressing both myosin heavy chains *MYH7* and *MYH2* (1/2A) and myofibers expressing neither *MYH7* nor *MYH2* (DN means double negative). Indeed, we found that *GADD45A* myofiber upregulation was associated with type 2A (*MYH2*-expressing) myofibers in IBM in contrast to other fiber types (Fig. 6d,e).

To summarize, spatial in situ validation based on snRNA-seq and ST revealed two myofiber damage pathways in IBM with cell/genomic stress markers associated with a selective type 2A myofiber pathology.

### Genomic stress and functional denervation in IBM

Electrophysiological evidence of myofiber denervation is commonly observed in electromyographic studies of individuals with IBM^31^. However, the mechanisms underlying myofiber denervation in inflamed muscles are not well understood. Based on previous reports it is known that Pumilio proteins are regulated by *NORAD*, among other genes^11,12^, and can regulate the translation of acetylcholinesterase (AChE)^32^. AChE, encoded by *ACHE*, is an enzyme best known for degrading acetylcholine at the NMJ of skeletal muscles. By ST analysis, we confirmed that *ACHE* expression was associated with the IBM-associated tissue niche 3 (Figs. 5d and 6f), and multiplex smFISH showed that *ACHE*^*hi*^/*NORAD*^*hi*^-coexpressing myofibers were enriched in IBM relative to CTRL and IMNM muscles (Fig. 6g,h). We next found that *COLQ*, encoding a protein critical for proper functioning of AChE at the NMJ^33^, was also upregulated in *ACHE*^*hi*^/*NORAD*^*hi*^-coexpressing myofibers in IBM (Fig. 6i,j) and confirmed that AChE protein expression was upregulated in IBM myofibers. Notably, we found no differences, but a trend toward an increase in NMJs in IBM relative to CTRL muscles (Fig. 6k, l).

Based on multiplex smFISH and IHC, we demonstrated that type 2A myofiber degeneration in IBM might be driven by functional denervation at the NMJ mediated by enhanced *NORAD* function, resulting in upregulation of AChE (Extended Data Fig. 6f).

## Discussion

Understanding the underlying processes contributing to inflammation and muscle atrophy in IBM is of prime interest in developing diagnostic biomarkers and targeted therapies. In the present study, we combined snRNA-seq and ST to study IIM pathology with a focus on IBM. We were able to confirm previous studies^34^ and observed a T cell-predominant inflammation paralleled by the presence of proinflammatory macrophages (that is, MΦ1) in IBM. Moreover, we observed signs of muscle tissue remodeling with a relative loss of type 1 MNs in both IIM subtypes, a specific loss of type 2 MNs and an increase of a presumably profibrotic cell state of *COL15A1*-expressing FAPs in IBM. Of note, marker genes associated with profibrotic FAPs such as *LOXL2* and *POSTN* have been reported in other fibrotic diseases^26–29^, and LOXL2 protein serum levels were increased in individuals with dermatomyositis^28^.

In addition, we found two specific MN subtypes, which we labeled as reactive MNs (present in IMNM and IBM) and damaged MNs (overrepresented in IBM). Based on gene expression, ‘reactive’ MNs showed signs of myofiber differentiation, regeneration and inflammation, but lacked signs of degeneration, hence representing an IIM-specific MN subtype at the crossroads between regeneration and degeneration. Conversely, ‘damaged’ *GADD45A/NORAD*-expressing MNs exhibited severe signs of myofiber atrophy and cell/genomic stress and were strongly associated with T cells. Enhanced *NORAD* activity has been reported under various conditions resulting in severe cell stress, for example, in damaged neurons in inflamed multiple sclerosis lesions^35^. We further noted that damaged MNs specifically upregulated *RNF7,* and RNF7 proteins colocalized with p62^+^ aggregates in myofibers in IBM. However, in contrast to p62^+^ myofibers, which have also been reported in other IIM subtypes including IMNM^36,37^, RNF7^+^ punctae appeared to be specific to IBM when compared with IMNM. Of note, GADD45A and RNF7 protein accumulation was spatially segregated and predominantly seen in different myofibers. Furthermore, GADD45A^+^ myofibers were specifically related to type 2A myofibers and spatially associated with T cell infiltrates as opposed to RNF7^+^ myofibers, suggesting different pathways of myofiber atrophy in IBM: (1) T cell-driven inflammation and genomic stress (GADD45A, lncRNA *NORAD*) and (2) degenerative processes involving protein degradation and autophagy (RNF7, p62). Moreover, it may also be possible that both pathways are not distinct, but rather a sequence of myofiber degeneration in IBM starting with infiltrating T cells leading to genomic stress, which later results in impaired protein degradation and autophagy.

In the present study, we put an emphasis on the genomic stress pathway and found that *ACHE* was upregulated in tissue niches containing damaged MNs. The translation of *ACHE* in myofibers is regulated by Pumilio proteins (especially PUM2), among others, which can withdraw *ACHE* transcripts from translation^32^. More specifically, Pumilio proteins affect the translation of their target proteins through different mechanisms, including inhibition of translation and acceleration of mRNA degradation^38^. *NORAD*, on the other hand, decreases the activity of Pumilio proteins, as mentioned above, and can therefore indirectly promote the translation of *ACHE* transcripts. An elevated expression of AChE might result in a lower concentration of acetylcholine at NMJs resulting in disrupted synaptic transmission and possible functional denervation and, as a consequence, fiber atrophy associated with the elevated expression of *GADD45A* in IBM^8,9^. Apart from being an essential part of neuromuscular transmission, AChE is also known to be involved in apoptosis by increasing the sensitivity of cells to apoptotic stimuli^39,40^. Moreover, it has been shown that apoptotic stressors can lead to increased AChE expression in human myoblasts and decreased AChE activity induced by specific small interfering RNA silencing can lead to decreased activation of caspases in myoblasts under apoptotic stress^41^. Hence, enhanced *ACHE* expression might trigger both functional fiber denervation (preferentially of type 2 fibers) and increase susceptibility to apoptosis.

In summary, the herein reported genomic stress pathway might represent a critical damage mechanism in IBM (in addition to degeneration through dysregulated protein degradation) and be a potential therapeutic target to improve NMJ transmission. Reversible AChE inhibitors have proved useful in treating various neurological conditions, with inhibitors crossing the blood–brain barrier (BBB, for example, rivastigmine) used in the therapy of dementia in Alzheimer’s and Parkinson’s diseases^42^ and ones not crossing the BBB (for example, pyridostigmine) used in the therapy of myasthenia gravis. Considering increased AChE expression in a subset of IBM myofibers, a non-BBB, crossing, reversible AChE inhibitor such as pyridostigmine might be a therapeutic consideration in IBM. Furthermore, lncRNAs such as *NORAD* have been investigated as potential targets for treating various cancers, because some lncRNAs have been shown to exhibit either tumor-suppressive or pro-oncogenic properties^43^. As we theorize that *NORAD* plays an important role in elevating AChE expression through inhibition of PUM2 activity, and considering the potential side effects of AChE inhibitors, targeting *NORAD* might prove more useful than targeting AChE itself. However, additional functional studies are needed to investigate the connection across *NORAD* and Pumilio activity, AChE upregulation and eventually myofiber atrophy as observed in IBM.

Moreover, as we hypothesize that type 2A myofibers might be more vulnerable to T cell-driven inflammation and genomic stress in IBM, improving resilience of type 2 fibers might be another promising strategy. Type 2 myofiber vulnerability is also a known pathological feature and therapeutic target in Duchenne muscular dystrophy (DMD) and Becker muscular dystrophy (BMD), where the focus is on a selective fast myosin inhibition to protect vulnerable fibers from mechanical stress. For example, fast myosin inhibition has proved beneficial in animal models suffering from muscular dystrophy, protecting skeletal muscles from stress injuries^44^. Specifically, the compound EDG-5506 was shown to reduce muscle damage biomarkers in adult patients with BMD^45^ and is currently in use in a phase 2 trial in children with DMD (NCT05540860). This shows that myofiber type-specific vulnerability is targetable and might be a promising therapeutic target in IBM as well, even if the mechanism leading to type 2 fiber vulnerability might differ.

With regard to immune cell heterogeneity in IBM, we found two subtypes of DCs (cDC1 cells and *LAMP3*-expressing DCs), which had not been described in IBM before. Of note, cDC1 cells are a subpopulation of DCs playing an important role in the activation of CTLs and T_H_1 cells^46,47^. Conversely, *LAMP3*-expressing DCs have been previously reported as mature and CCR7-dependent migratory DCs with the ability to travel to adjacent lymph nodes^48,49^. Considering the interactions of cDC1 cells with T cells and the interactions of DCs with myoblasts inducing their proliferation^50^, DCs represent another target cell of interest in IBM. In addition to adaptive immune cells, we noted that homeostatic and probably regulatory macrophages (MΦ2 cells) were reduced in IBMs, suggesting that a loss of regulatory immune cell function might be a critical driver of tissue inflammation in IBM. Considering endothelial and stromal cells, we observed a relative loss of EC-C cells in IBM which, to our knowledge, has not been reported earlier. Previous studies suggested that the capillary density in IBM is not reduced in contrast to the reduced density in dermatomyositis^51^, but reported that the microvascular architecture seems to be distorted in IBM^52^. Therefore, we assume that the herein observed loss is the result of ongoing tissue remodeling in IBM with loss of myofibers, resulting in lower numbers of capillaries, because myofibers and capillaries in skeletal muscle are closely connected. Moreover, higher counts of FAPs might also reduce the proportion of EC-C cells within populations of endothelial and stromal cells.

As IBM is a slowly progressive muscle disease, it is not possible to precisely determine the beginning of the disease. Hence, the disease duration at the point of biopsy remains unknown. Therefore, we can provide only a snapshot of the cell-type-specific molecular changes underlying IBM pathogenesis and future studies will be needed to determine whether the herein reported marker genes and pathways can be used as therapeutic targets and biomarkers to stratify individuals into clinical subtypes with selective risk levels regarding clinical progression.

## Methods

### Muscle biopsies

Institutional review board (IRB) approvals (see below) permitting the use of the taken biopsies for research purposes were in place at all donating sites. There was no participant compensation. Noninflammatory controls and IBM samples were obtained from the Institute of Neuropathology, University Medical Center of the Johannes Gutenberg-University Mainz (Germany, IRB approval no. 2020-15215_1); noninflammatory controls and IBM samples were obtained from the Department of Neurology, Ulm University (Germany, IRB approval no. 20/10); noninflammatory controls, anti-signal recognition particle (SRP) IMNM and IBM samples were obtained from the Johns Hopkins Myositis Center (IRB approval nos. IRB00235256 and IRB00072691), Baltimore (MA, USA) and the Department of Neuropathology at the Charité University Medical Center, Berlin (Germany, IRB approval no. EA2/163/17). All samples were taken from patients for diagnostic purposes and stored afterwards at −80 °C. Muscle biopsies were obtained following written informed consent by the donors. Overall, a total of 29 noninflammatory controls, 13 IMNM and 37 IBM samples were available for this study.

### Histochemistry, IHC and in situ hybridization

Samples were sectioned at 10 µm in a C3050S Cryostat (Leica Microsystems) and mounted on SuperFrost Slides (VWR). Performed histochemical staining techniques were hematoxylin and eosin (H&E) and modified Gömöri’s trichrome. Fluorescent and chromogenic IHC were performed as previously described^53^ and the following primary antibodies were used: anti-*GADD45A* (OriGene, cat. no. TA507370, 1:1,000), anti-RNF7 (Proteintech, cat. no. 11905-1-AP, 1:1,000), anti-CD3 (BioRad, cat. no. MCA772, 1:200), anti-p62/SQSTM1 (Santa Cruz Biotechnology, cat. no. sc-28359, 1:1,000), anti-AChE (Abcam, cat. no. ab183591, 1:100) and anti-laminin (Santa Cruz Biotechnology, cat. no. sc-59854, 1:100), which were diluted with the blocking solutions. With regard to secondary antibodies, we used biotinylated goat anti-mouse immunoglobulin G (IgG) (Vector Laboratories, cat. no. BA-9200-1.5; Thermo Fisher Scientific, cat. no. 62-6540, each 1:500), Alexa Fluor-488 goat anti-rabbit IgG (Thermo Fisher Scientific, cat. no. A-11034, 1:500), Alexa Fluor-488 goat anti-rat IgG (Thermo Fisher Scientific, cat. no. A-11006, 1:500), Alexa Fluor-488 goat anti-mouse IgG1 (Thermo Fisher Scientific, cat. no. A-21121 1:500), Alexa Fluor-555 goat anti-rabbit IgG (Thermo Fisher Scientific, cat. nos. A-21428 and A-32732, each 1:500), Alexa Fluor-555 goat anti-mouse IgG2b (Thermo Fisher Scientific, cat. no. A-21147, 1:500), and Alexa Fluor-647 goat anti-rat IgG (Thermo Fisher Scientific, cat. no. A-21247, 1:500).

SmFISH was performed using the ACD RNAscope Multiplex Assay v2 according to provided protocols using the fluorophores FITC, Cy3 and Cy5. The following RNA probes were used: Hs-3-plex positive control (ACD 320861), 3-plex negative control (ACD 320871), Hs-*GADD45A* (ACD 477511), Hs-*NORAD* (ACD 525631), Hs-*ACHE* (ACD 519271), Hs-*COLQ* (ACD 584691), Hs-*MYH2* (ACD 504731) and Hs-*MYH7* (ACD 508201).

### RNA quality control, nuclei isolation and snRNA-seq

For RNA isolation, 50-µm-thick cryosections were cut to obtain 10–20 mg of tissue, which was then homogenized in TRIzol (Thermo Fisher, cat. no. 15596018), centrifuged with chloroform and purified using the RNeasy Mini kit (QIAGEN, cat. no. 74104). To determine RNA integrity, we used the High Sensitivity RNA assay (Agilent, cat. nos. 5067-5579, 5067-5580 and 5067-5581) on an Agilent 4200 TapeStation System. Samples with an RNA integrity number (RIN) < 7 were excluded from snRNA-seq and ST experiments.

For nuclei isolation, we used a modified protocol similar to the one previously described^35^. In the present study, 20–30 mg of muscle tissue was chopped and homogenized using 2 ml of lysis buffer (0.32 M sucrose, 5 mM CaCl_2_, 3 mM Mg(Ac)_2_, 0.1 mM EDTA, 10 mM Tris-HCl, 1 mM dithiothreitol (DTT), 0.5% Triton X-100 in diethyl pyrocarbonate (DEPC)-treated water) and a glass Dounce homogenizer with a tight pestle on ice. After homogenization the lysate was transferred into a 17-ml poly(propylene) ultracentrifuge tube and 3.7 ml of sucrose buffer (1.8 M sucrose, 3 mM Mg(Ac)_2_, 10 mM Tris-HCl, 1 mM DTT in DEPC-treated water) was pipetted below, subsequently filled up with lysis buffer and then centrifuged at 107,163.6*g* for 2.5 h at 4 °C. Afterwards, the supernatant was removed and the nuclei pellet was incubated in 200 µl of DEPC-treated, water-based 1× phosphate-buffered saline for 20 min on ice and then resuspended. After filtering with 30-µm filters, the nuclei suspension was examined by light microscopy for the presence of debris, then nuclei were manually counted using a hemocytometer and loaded on to a Chromium X Series controller (10x Genomics) aiming for 5,000 nuclei per sample. Libraries were generated according to the 10x Next GEM 3′ Kit v.3.1 protocol (10x Genomics, CG000204, rev. nos. D/PN-1000121, PN-1000127 and PN-1000213). After recommended QC, all libraries were pooled and sequenced on an Illumina NovaSeq 6000 system. Sequencing was performed at the Institute of Clinical Biology (IKMB, Kiel).

### PC and QC analysis workflow

Nuclei Fastq files were trimmed and aligned to the human GRCh38-2020-A transcriptome using the cellranger (v.4.0.0) count function. QC, normalization and dimensionality reduction were performed on each sample separately using the R package Seurat (v.4.1.0)^54^. Nuclei with <700 counts and >5% of mitochondrial genes were defined as bad-quality nuclei and were excluded from downstream analysis. Data were normalized using SCTransform^55^ as implemented by Seurat. For the selection of principal components (PCs), we first calculated the variance explained by each PC and defined the first cutoff as the number of PCs with a cumulative variance of >90%. Then, we determined the second PC cutoff so that the variance of consecutive PCs was <10%. The number of PCs was defined as a minimum of both above-mentioned cutoffs. Visualization and clustering were performed using built-in functions in Seurat: RunUMAP(), FindNeighbors() and FindClusters() with default parameters. Next, low-quality clusters were identified using the built-in function FindAllMarkers() by displaying no marker genes, mitochondrial encoded genes, ribosomal genes or genes suggesting the presence of doublets that were then removed. After excluding all low-quality nuclei, background mRNA contamination was quantified and removed using the R package SoupX (v.1.5.2)^56^ with default parameters. All droplets (including those that did not pass QC) were used for estimating background contamination. Estimated background contamination was removed from nuclei that have passed QC. After excluding low-quality cells and removing background mRNA contamination, mitochondrial genes were excluded from counts.

### Computational data analysis and integration

Further analysis was performed using Python (v.3.9.2). Filtered counts were loaded using scanpy (v.1.7.2) and a second QC was performed, keeping counts between 1,000 and 20,000 reads per nucleus and nuclei with >800 genes^57^. Nuclei with >2% of mitochondrial DNA were excluded and doublet detection was performed using scrublet (v.0.2.3) with a threshold cutoff of 0.25 for predicted doublet scores for each sample individually^58^. Samples were merged using sc.concat command. Normalized log-transformed counts were calculated using the default scanpy functions and 2,500 highly variable genes were filtered for clustering using default parameters of the pp.highly_variable_genes command. The number of counts per nucleus and mitochondrial percentage counts were regressed out (sc.pp.regress_out), counts were scaled to a maximum value of 10 and integration was performed using BBKNN (v.1.5.1) with ridge regression enabled, considering a number of 25 PCs^59^. Clustering was performed using the Leiden algorithm with 0.5 resolution. Plotting of Uniform Manifold Approximations and Projections (UMAPs), dot plots and violin plots were done using scanpy functions. Marker genes per cluster were identified using Wilcoxon’s rank-sum test comparing each cluster against the rest of the clusters combined. Bar plots for compositional analysis were generated using sccoda (v.0.1.7) with default parameters^60^. Statistical analysis of cell type proportions (compositional analysis) and box -plot generation was performed using R (v.4.0.5) in RStudio (v.2022.02.3+492) using the following packages: stats (v.4.0.5), ggplot2 (v.3.3.2) and tidyverse (v.1.1.0)^61–64^. Subclusters were subsetted from raw counts and processed as described above. The number of PCs used was chosen to explain >90% of variance in the subset. Clustering resolutions were chosen according to the complexity of the subset (myonuclei subset 0.3, immune cell subset 0.4, endothelial–stromal cell subset 0.3). GSEA was performed using gseapy (v.1.0.4)^65^. Subsets of interest were generated by subsetting normalized log-transformed counts. The GO biological processes 2021 gene set was selected; however, genes that did not exist in the transcriptome were removed from the gene set to consider the background information. Gene set enrichment was performed with the recommended setting of 1,000 permutations of the phenotype sort and the signal-to-noise method was used. Plotting was performed with the gseapy plot.dotplot function on the top seven up- and downregulated terms, chosen by their normalized expression score (NES), as long as their false recovery rate (FDR) was <0.25.

Differential abundance analysis was performed using miloR (v.1.6.0)^24^ in R (v.4.2.2). In the present study, retrieved nuclei were organized into partially overlapping neighborhoods based on a computed *k*-nearest neighbor (KNN) graph. Specifically, we converted the h5ad objects from each subclustering analysis into a SingleCellExperiment object using rpy2 (v.3.5.7) and analyzed the data according to the miloR workflow as described in its vignettes. As recommended, we passed the data sequentially to the testNhoods() function and performed an additional correction to the spatial FDR values using the Benjamini–Hochberg correction to account for multiple comparisons. Neighborhoods with a corrected spatial FDR <0.1 were considered differentially abundant. The neighborhoods found in endothelial–stromal cells were then subdivided into groups based on the number of overlapping cells between neighborhoods and the direction of log(fold change) of abundances between CTRLs and IBM. In the present study, neighborhoods with a spatial FDR < 0.1 were considered differentially abundant.

### Spatial transcriptomics

The 10x Genomics Visium Spatial Gene Expression platform was used for the spatial transcriptomics experiments. In short, 10x Genomics Visium Spatial Gene Expression slides have four capture areas (6.5 × 6.5 mm^2^), each defined by a fiducial frame (8 × 8 mm^2^). Each capture area has an array of approximately 5,000 circular spots with primers that include an Illumina TruSeq Read 1 (partial read 1 sequencing primer), 16-nt spatial barcode (all primers in a specific spot will share the same barcode), 12-nt unique molecular identifier (UMI) and 30-nt poly(dT) sequences that capture poly(adenylated) mRNA for complementary DNA synthesis. The tissue (RIN ≥ 7) was cut into 10-µm sections using a Leica CM3050 S cryostat and placed onto Spatial Gene Expression Slides (cat. no. PN-1000185) that were precooled inside the cryostat at −28 °C. The slides were stored in a container at −80 °C until further processing. The sections were then fixed and stained (H&E) following the protocol CG000160 Rev B and imaged using the ×10 brightfield objective from the Leica DMi8 and processed by the Leica Application Suite X (LAS X). After being imaged to do a general morphological analysis and for future spatial alignment of the data, the samples were enzymatically permeabilized for 18 min. This time was assessed using the 10x Visium Tissue Optimization kit (cat. no. PN-1000191) following the protocol CG000238 Rev D. The generation of the libraries was performed according to protocol CG000239 Rev D, using the Gene Expression Reagent kit (cat. no. PN-1000186), the Library Construction kit (cat. no. PN-1000190) and the Dual Index Plate TT Set A (cat. no. PN-1000215). To assess the correct amplification of the cDNA, QuantStudio 3 from Thermo Fisher Scientific was used. For full-length cDNA and indexed library analysis, the 4200 TapeStation System from Agilent was used. The seven libraries were loaded at 300 pM and sequenced on a NovaSeq 6000 System from Illumina with a sequencing depth of 250 million reads per sample. Sequencing was performed at the IKMB (Kiel). The demultiplexing of the data was done using spaceranger software (v.2.0.0), creating Fastq files. These files are then used by spaceranger count to perform alignment with the human reference transcriptome (GRCh38-2020-A), tissue detection, fiducial detection and barcode/UMI counting.

Counts per sample were loaded into scanpy and spots with <600 counts and 200 genes were excluded. Genes that were present in <10 spots were also excluded from further analysis^57^. After samples were merged, a counts per million normalization was performed and log-transformed counts were calculated. The merged samples were integrated using BBKNN with ridge regression and ten PCs included^59^. The merged object was clustered using the Leiden algorithm at resolution 0.5 and plotted using pl.umap and pl.stacked_violin functions in scanpy. Spatial data was deconvoluted using cell2location v.0.1 with 10 cells per location and a detection α of 200 following the recommended script^66^. Unnormalized, single-cell data with cluster information were used as a reference and q05 abundance counts were used for downstream analysis. Correlations between cell type abundances were calculated using the scipy Pearson’s correlation coefficient. Mean cell type abundance per niche was calculated and scaled using sklearn StandardScaler and heatmaps were visualized using matplotlib.pyplot. Furthermore, neighborhood enrichment was calculated using native squidpy functions gr.spatial_neighbors and gr.nhood_enrichment^67^. GSEA was performed as described earlier, with the difference that here the given Leiden clustering was used as categorization. Niche 3 was tested versus the rest of all other niches combined.

### Microscopy and quantitative image analysis

Images were taken with ×10, ×20 or ×40 objectives using a Leica DMi8 widefield microscope (Leica DFC7000 GT and DMC 4500 camera), a Leica DM6 B microscope (Leica K3C camera), a Leica TCS SP8 laser confocal microscope (405/488/552/638 mm) or a Keyence (BZ-X700) widefield microscope. Fluorescent images were taken using *z*-stack steps.

Images were loaded into Fiji ImageJ (v.2.1) for image processing and quantitative analysis. Positive cells/myofibers were counted and areas of whole sections were measured in Fiji to determine positive cells/myofibers per mm^2^. We chose a different approach to evaluate the CD3^+^ T cell infiltration associated with GADD45A and RNF7 expression, respectively. There, we chose ten GADD45A^+^RNF7^−^ and five to ten GADD45A^−^RNF7^+^ myofibers per sample in eight samples, having the low RNF7 expression rate in general as a limiting factor, and quantified the number of CD3^+^ T cells within a circle with a radius of 100 μm, setting the center of the circle in the center of the corresponding fiber. To exclude GADD45A^+^p62^+^ myofibers from analysis, we checked for p62 aggregates on consecutive slides. Besides, we chose only fibers with no other neighboring GADD45A^+^ and/or RNF7^+^ myofibers, so that the T cell infiltration could be attributed to one myofiber only. Afterwards, we calculated the average number of T cells surrounding GADD45A^+^RNF7^−^ and GADD45A^−^RNF7^+^ myofibers per sample.

Values were transferred into Excel sheets and statistical analysis and plot generation were performed using R (v.4.0.5) in RStudio (v.2022.02.3+492) and the following packages: stats (v.4.0.5), ggplot2 (v.3.3.2) and tidyverse (v.1.1.0)^61–64^.

### Statistics and reproducibility

No statistical method was used to predetermine sample size, but sample sizes are similar to those reported previously^35,68,69^. Nuclei (snRNA-seq) and spots (ST) of low quality, which did not pass QC (see above), were excluded. The experiments were not randomized. The investigators were not blinded to allocation during experiments and outcome assessment. H&E and modified Gömöri’s trichrome stainings were performed on all samples included in the present study with Fig. 1b showing representative images of the three conditions. A histochemical staining (in this case H&E; see above) is part of the ST protocol that we used. Extended Data Fig. 2a shows all H&E stainings of the sequenced ST samples (*n* = 8). Extended Data Fig. 6e shows representative images of IHC performed on CTRL and IMNM muscle. To determine differences between groups in compositional analysis and validation via IHC or smFISH, we first checked whether groups were normally distributed by using the Shapiro–Wilk test. If normality was confirmed, we used Bartlett’s test to check for homogeneous variances. For comparisons of multiple groups, we used one-way analysis of variance followed by Tukey’s honestly significant difference (HSD) test if normality and homogeneous variances were confirmed; otherwise the Kruskal–Wallis test was used followed by pairwise Wilcoxon’s rank-sum tests (two-tailed) and *P*-value adjustment using the Benjamini–Hochberg correction. For comparison of paired groups, we used paired Student’s *t*-tests (two-tailed) or Wilcoxon’s signed-rank test (two-tailed), depending on normality, and applied the Benjamini–Hochberg correction for *P*-value adjustment in case of multiple comparisons. The following significance levels were used: ^*^P < 0.05, ^**^P < 0.01,^***^P < 0.001.

### Reporting summary

Further information on research design is available in the Nature Portfolio Reporting Summary linked to this article.

### Supplementary information

Reporting Summary

Supplementary Tables 1–15: Supplementary Table 1 Additional information on samples used in the present study. Supplementary Table 2 Clinicopathological data on sequenced samples. Supplementary Table 3 Top 50 marker genes for clusters of all captured nuclei identified by snRNA-seq. Wilcoxon’s rank-sum tests were performed to calculate *P* values followed by Benjamini–Hochberg correction to account for multiple comparisons. Supplementary Table 4 Top 50 marker genes for clusters of all captured myonuclei identified by snRNA-seq. Wilcoxon’s rank-sum tests were performed to calculate *P* values followed by Benjamini–Hochberg correction to account for multiple comparisons. Supplementary Table 5 Top 50 marker genes for clusters of all captured immune cell nuclei identified by snRNA-seq. Wilcoxon’s rank-sum tests were performed to calculate *P* values followed by Benjamin–Hochberg correction to account for multiple comparisons. Supplementary Table 6 Top 50 marker genes for clusters of all captured endothelial-stromal cells identified by snRNA-seq. Wilcoxon’s rank-sum tests were performed to calculate *P* values followed by Benjamini–Hochberg correction to account for multiple comparisons. Supplementary Table 7 Up- and downregulated gene sets in type 1 MNs compared between IBM and CTRLs. The ‘signal-to-noise’ method was used as a statistical test for data analysis. Supplementary Table 8 Up- and downregulated gene sets in type 2 MNs compared between IBM and CTRLs. The ‘signal-to-noise’ method was used as a statistical test for data analysis. Supplementary Table 9 Up- and downregulated gene sets in type 1 MNs compared between IBM and IMNM. The ‘signal-to-noise’ method was used as a statistical test for data analysis. Supplementary Table 10 Up- and downregulated gene sets in type 2 MNs compared between IBM and IMNM. The ‘signal-to-noise’ method was used as a statistical test for data analysis. Supplementary Table 11 Up- and downregulated gene sets in type 1 MNs compared between IMNM and CTRLs. The ‘signal-to-noise’ method was used as a statistical test for data analysis. Supplementary Table 12 Up- and downregulated gene sets in type 2 MNs compared between IMNM and CTRLs. The ‘signal-to-noise’ method was used as a statistical test for data analysis. Supplementary Table 13 Top marker genes for all identified groups of endothelial–stromal cells identified by miloR. *P* values were calculated using a moderated Student’s *t*-test and adjusted using the Benjamini–Hochberg correction to account for multiple comparisons. Supplementary Table 14 Top 50 marker genes for tissue niches identified by ST. Wilcoxon’s rank-sum tests were performed to calculate *P* values followed by the Benjamini–Hochberg correction to account for multiple comparisons. Supplementary Table 15 Up- and downregulated gene sets in niche 3 compared with the other tissue niches. The ‘signal-to-noise’ method was used as a statistical test for data analysis.

## Data Availability

SnRNA-seq and ST datasets were uploaded and are available for download and as an interactive cell browser (https://muscle-ibm.cells.ucsc.edu). Raw sequencing data (Fastq files) are available at the Human Cell Atlas Data Coordination Platform (project UUID: d5c91e92-2e7f-473d-8cf3-ab03bbae21c2) and have been deposited in the European Genome Archive repository: accession no. EGAS50000000310. The GRCh38-2020-A reference transcriptome was used for snRNA-seq and ST data analysis. All other data supporting the findings of the present study are available from the corresponding authors upon request.

## Code availability

Customized codes are available at the following link: https://github.com/schirmerlab/IBMseq.
