# Supplementary material for: Cell type mapping of inflammatory muscle diseases highlights selective myofiber vulnerability in inclusion body myositis
Source: Nat Aging. 2024 Jun 4;4(7):969–83. doi: 10.1038/s43587-024-00645-9 (PMC11257986; doi:10.1038/s43587-024-00645-9)
Supplement: Supplementary file 1 — Reporting Summary [file 43587_2024_645_MOESM1_ESM.pdf]

nature portfolio

Corresponding author(s): Lucas Schirmer, Thomas E Lloyd

Last updated by author(s): Apr 23, 2024

Reporting Summary

Nature Portfolio wishes to improve the reproducibility of the work that we publish. This form provides structure for consistency and transparency in reporting. For further information on Nature Portfolio policies, see our Editorial Policies and the Editorial Policy Checklist.

Statistics

For all statistical analyses, confirm that the following items are present in the figure legend, table legend, main text, or Methods section.

n/a	Confirmed	
☐	☒	The exact sample size (*n*) for each experimental group/condition, given as a discrete number and unit of measurement
☐	☒	A statement on whether measurements were taken from distinct samples or whether the same sample was measured repeatedly
☐	☒	The statistical test(s) used AND whether they are one- or two-sided<br>*Only common tests should be described solely by name; describe more complex techniques in the Methods section.*
☒	☐	A description of all covariates tested
☐	☒	A description of any assumptions or corrections, such as tests of normality and adjustment for multiple comparisons
☐	☒	A full description of the statistical parameters including central tendency (e.g. means) or other basic estimates (e.g. regression coefficient) AND variation (e.g. standard deviation) or associated estimates of uncertainty (e.g. confidence intervals)
☐	☒	For null hypothesis testing, the test statistic (e.g. F, t, r) with confidence intervals, effect sizes, degrees of freedom and P value noted<br>*Give P values as exact values whenever suitable.*
☒	☐	For Bayesian analysis, information on the choice of priors and Markov chain Monte Carlo settings
☒	☐	For hierarchical and complex designs, identification of the appropriate level for tests and full reporting of outcomes
☐	☒	Estimates of effect sizes (e.g. Cohen's *d*, Pearson's *r*), indicating how they were calculated

Our web collection on statistics for biologists contains articles on many of the points above.

Software and code

Policy information about availability of computer code

Data collection	Geneset resources for gene set enrichment analysis (GSEA) were fetched from the python package gseapy v1.0.4.
Data analysis	Cellranger v4.0.0, SpaceRanger v2.0.0, R v4.0.5, RStudio v2022.02.3, stats v4.0.5, ggplot2 v3.3.2, tidyverse v1.1.0 Seurat v4.1.0, SoupX v1.5.2, Python v3.9.2, scrublet v0.2.3, scanpy v1.7.2, BBKNN v1.5.1, sccoda v0.1.7, squidpy v1.2.2, gseapy v1.0.4, cell2location v0.1, rpy2 v3.5.7, R v4.2.2, miloR v1.6.0, and Fiji ImageJ v2.1

For manuscripts utilizing custom algorithms or software that are central to the research but not yet described in published literature, software must be made available to editors and reviewers. We strongly encourage code deposition in a community repository (e.g. GitHub). See the Nature Portfolio guidelines for submitting code & software for further information.

Data

Policy information about availability of data

All manuscripts must include a data availability statement. This statement should provide the following information, where applicable:

- Accession codes, unique identifiers, or web links for publicly available datasets
- A description of any restrictions on data availability
- For clinical datasets or third party data, please ensure that the statement adheres to our policy

snRNA-seq and ST data sets were uploaded and are available for download and as an interactive cell browser (https://muscle-ibm.cells.ucsc.edu). Raw sequencing data (Fastq files) are available at the Human Cell Atlas Data Coordination Platform (HCA DCP) (project UUID: d5c91e92-2e7f-473d-8cf3-ab03bbae21c2) and have

been deposited in the European Genome Archive (EGA) repository: EGAS50000000310. The GRCh38-2020-A reference transcriptome was used for snRNA-seq and ST data analysis. All other data supporting the findings of this study are available from the corresponding authors upon request.

Research involving human participants, their data, or biological material

Policy information about studies with human participants or human data. See also policy information about sex, gender (identity/presentation), and sexual orientation and race, ethnicity and racism.

Reporting on sex and gender	Sex was determinded based on self-reporting, information on gender was not collected. For snRNA-seq studies, 37% of the donors were female, 63% were male (snRNA-seq), and for ST studies 38% of the donors were female, 62% were male.
Reporting on race, ethnicity, or other socially relevant groupings	Information on race, ethnicity or other socially relevant groupings was not available due to anonymization.
Population characteristics	snRNA-seq:<br>CTRL: n = 7 (5 male, 2 female), mean age = 64 years; IMNM: n = 4 (2 male, 2 female), mean age = 59 years; IBM: n = 8 (5 male, 3 female), mean age = 68 years<br><br>ST:<br>CTRL: n = 3 (2 male, 1 female), mean age = 67 years; IMNM: n = 2 (1 male, 1 female), mean age = 58 years; IBM: n = 3 (2 male, 1 female), mean age = 67 years<br><br>ISH (ACHE/GADD45A/NORAD + MYH7/GADD45A/COLQ):<br>CTRL: n = 5 (3 male, 2 female), mean age = 62 years; IMNM: n = 4 (3 male, 1 female), mean age = 60 years; IBM: n = 5 (4 male, 1 female), mean age = 68 years.<br><br>ISH (GADD45A/NORAD):<br>CTRL: n = 5 (3 male, 2 female), mean age = 62 years; IMNM: n = 4 (3 male, 1 female), mean age = 60 years; IBM: n = 9 (7 male, 2 female), mean age = 66 years<br><br>ISH (MYH7/GADD45A/MYH2):<br>IBM: n = 7 (5 male, 2 female), mean age = 66 years<br><br>IHC (GADD45A):<br>CTRL: CTRL: n = 12 (8 male, 4 female), mean age = 56 years; IMNM: n = 12 (7 male, 5 female), mean age = 55 years; IBM: n = 28 (20 male, 8 female), mean age = 68 years<br><br>IHC (GADD45A%):<br>CTRL: n = 6 (5 male, 1 female), mean age = 57 years; IMNM: n = 8 (4 male, 4 female), mean age = 53 years; IBM: n = 20 (14 male, 6 female), mean age = 69 years<br><br>IHC (RNF7):<br>CTRL: n = 11 (8 male, 3 female), mean age = 59 years; IMNM: n = 12 (7 male, 5 female), mean age = 55 years; IBM: n = 26 (19 male, 7 female), mean age = 69 years<br><br>IHC (CD3/GADD45A/RNF7):<br>IBM: n = 8 (7 male, 1 female), mean age = 67 years<br><br>IHC (RNF7 colocalizations):<br>IBM: n = 6 (5 male, 1 female), mean age = 70 years<br><br>IHC (ACHE):<br>CTRL: n = 20 (11 male, 9 female), mean age = 56 years; IMNM: n = 8 (4 male, 4 female), mean age = 53 years;<br>IBM: n = 23 (15 male, 8 female), mean age = 70.
Recruitment	Human muscle biopsies were provided by the University Medical Center of the Johannes Gutenberg-University Mainz (Institute of Neuropathology), the Johns Hopkins University School of Medicine, Baltimore (Johns Hopkins Myositis Center), Ulm University (Department of Neurology) and the Charité University Medical Center Berlin (Department of Neuropathology).
Ethics oversight	Muscle biopsies were obtained following written informed consent by the donors following ethical approvals by the University Medical Center of the Johannes Gutenberg-University Mainz (Germany, institutional review board approval 2020-15215_1), the Department of Neurology, Ulm University (Germany, institutional review board approval 20/10), the Johns Hopkins Myositis Center (institutional review board approval IRB00235256 and IRB00072691), Baltimore (Maryland, USA) and the Department of Neuropathology at the Charité University Medical Center, Berlin (Germany, institutional review board approval EA2/163/17).

Note that full information on the approval of the study protocol must also be provided in the manuscript.

Field-specific reporting

Please select the one below that is the best fit for your research. If you are not sure, read the appropriate sections before making your selection.

☒ Life sciences ☐ Behavioural & social sciences ☐ Ecological, evolutionary & environmental sciences

For a reference copy of the document with all sections, see nature.com/documents/nr-reporting-summary-flat.pdf

Life sciences study design

All studies must disclose on these points even when the disclosure is negative.

Sample size	Sample size was determined based on tissue availability, tissue quality and (in case of sequencing studies) RNA quality. No sample size calculation was performed, but sample sizes are similar to previous studies (see "Methods" section "Statistics and reproducibility").
Data exclusions	Samples were excluded from sequencing studies if they did not meet the minimum RNA quality as described in the methods section. Further, sequenced nuclei (snRNA-seq) and spots (ST) of low quality, which did not pass quality control as described in the "Methods" section, were excluded.
Replication	Transcriptomic data from thousands of cells (snRNA-seq) and spots (ST) were collected and analyzed for each sample and condition. For IHC and smFISH, several biological replicates were used of each condition.
Randomization	No randomization was applied. Human muscle biopsies were catagorized by clinical/histopathological diagnosis.
Blinding	Unsupervised leiden clustering algorithm was used during transcriptomic analysis. Due to obvious histopathological changes in the tissue immunohistochemical and in situ hybridization analyses could not be performed blinded.

Reporting for specific materials, systems and methods

We require information from authors about some types of materials, experimental systems and methods used in many studies. Here, indicate whether each material, system or method listed is relevant to your study. If you are not sure if a list item applies to your research, read the appropriate section before selecting a response.

Materials & experimental systems

n/a	Involved in the study
☐	☒ Antibodies
☒	☐ Eukaryotic cell lines
☒	☐ Palaeontology and archaeology
☒	☐ Animals and other organisms
☒	☐ Clinical data
☒	☐ Dual use research of concern
☒	☐ Plants

Methods

n/a	Involved in the study
☒	☐ ChIP-seq
☒	☐ Flow cytometry
☒	☐ MRI-based neuroimaging

Antibodies

Antibodies used	Primary antibodies: anti-GADD45A (OriGene TA507370), anti-RNF7 (Proteintech 11905-1-AP), anti-CD3 (BioRad MCA772), anti-p62/SQSTM1 (Santa Cruz Biotechnology sc-28359), anti-ACHE (Abcam ab183591), anti-Laminin (Santa Cruz Biotechnology sc-59854)<br>Secondary antibodies: Biotinylated goat anti-mouse IgG (Vector Laboratories, BA-9200-1.5; and Thermo Fisher, 62-6540), Alexa Fluor 488 goat anti-rabbit IgG (ThermoFisher, A-11034), Alexa Fluor 488 goat anti-rat IgG (ThermoFisher, A-11006), Alexa Fluor 488 goat anti-mouse IgG1 (ThermoFisher, A-21121), Alexa Fluor 555 goat anti-rabbit IgG (ThermoFisher, A-21428 and A32732), Alexa Fluor 555 goat anti-mouse IgG2b (ThermoFisher, A-21147), and Alexa Fluor 647 goat anti-rat IgG (ThermoFisher, A-21247)
Validation	All listed antibodies are commercially available.<br>anti-GADD45A antibody was validated by manufacturer for Western Blot (human)<br>PMID: 32896253 for anti-p62/SQSTM1 antibody (IHC), recommended by manufacturer for IHC (human)<br>PMID: 36476314 for anti-Laminin antibody (IHC), recomemended by manufacturer for IHC (human)<br>PMID: 30844312 for anti-RNF7 antibody (Western Blot), validated by manufacturer for Western Blot in human skeletal muscle and IF in mouse heart muscle<br>PMID: 31176305 for anti-ACHE antibody (IHC, species: rat), validated by manufacturer for WB, IHC-Fr, IHC-P (mouse and rat)

Plants

Seed stocks

Report on the source of all seed stocks or other plant material used. If applicable, state the seed stock centre and catalogue number. If plant specimens were collected from the field, describe the collection location, date and sampling procedures.

Novel plant genotypes

Describe the methods by which all novel plant genotypes were produced. This includes those generated by transgenic approaches, gene editing, chemical/radiation-based mutagenesis and hybridization. For transgenic lines, describe the transformation method, the number of independent lines analyzed and the generation upon which experiments were performed. For gene-edited lines, describe the editor used, the endogenous sequence targeted for editing, the targeting guide RNA sequence (if applicable) and how the editor was applied.

Authentication

Describe any authentication procedures for each seed stock used or novel genotype generated. Describe any experiments used to assess the effect of a mutation and, where applicable, how potential secondary effects (e.g. second site T-DNA insertions, mosiacism, off-target gene editing) were examined.
